# Enhancing CAR-NK persistence to unlock its full therapeutic potentials

**DOI:** 10.3389/fimmu.2026.1870668

**Published:** 2026-07-08

**Authors:** Ovini Amarasinghe, Heqing Ma, Cédric S. Tremblay, Sam K. P. Kung

**Affiliations:** 1 Department of Immunology, Max Rady College of Medicine, Rady Faculty of Health Sciences, University of Manitoba, Winnipeg, MB, Canada; 2 Paul Albrechtsen Research Institute CancerCare Manitoba, Winnipeg, MB, Canada; 3 Children’s Hospital Research Institute of Manitoba (CHRIM), Winnipeg, MB, Canada

**Keywords:** CAR, genetic engineering, NK, persistence, tumor immunotherapy

## Abstract

Chimeric Antigen Receptor-Natural Killer Cell (CAR-NK) is a promising next-generation immunotherapy. Persistence of CAR-NK cell therapy is considered a key hurdle limiting its full therapeutic potential. This review highlighted the causes of poor CAR-NK persistence and summarized strategies developed to improve CAR-NK persistence in both hematologic malignancies and solid tumors. These strategies included cytokine and cytokine signaling mediated strategies; optimization of NK cell source and CAR design; intrinsic and extrinsic checkpoint disruption and metabolic reprogramming; and lymphodepletion, alloevasion and fratricide evasion strategies. Multiple strategies that aim at improving *in vivo* persistence should prove useful in enhancing therapeutic efficacy of CAR-NK.

## 1 Introduction

Cellular immunotherapy has transformed cancer treatment. Development of Chimeric Antigen Receptor (CAR) technology in CAR-T immunotherapy has demonstrated clinical successes, especially in the treatment of hematologic malignancies (HM). However it has also revealed challenges which spurred increasing focus on CAR-Natural Killer (CAR-NK) cells (1). CAR-NK is an attractive next generation immunotherapy over CAR-T as: 1) lacking a T-Cell Receptor (TCR) allows allogenic use without risking Graft-versus-Host Diseases (GvHD), enabling scalable “off-the-shelf” manufacturing; 2) the ability to kill cancer cells in both a CAR-dependent and independent manner; 3) lowering incidence of Cytokine Release Syndrome (CRS) and neurotoxicity which makes them safer; 4) less prone to differentiation-induced functional changes, 5) reduced on-target, off-tumor toxicity, and 6) relatively less intensive preconditioning before treatment (1–5).

CAR-NK therapies have advanced from preclinical models into clinical trials, highlighting immense research interests and promises in this field (6). Key challenges to overcome for unleashing their full efficacies in immunotherapy: 1) immunosuppressive tumor microenvironments that dampen CAR-NK activity; 2) restricted trafficking and infiltration into solid tumors; 3) susceptibility to NK cell exhaustion; 4) tumor antigen heterogeneity and escape; and short *in vivo* persistence (1, 7, 8). Unmodified CAR-NK cells have limited persistence of approximately 1–4 weeks (9–12). This review summarizes the main causes underlying CAR-NK persistence *in vivo*, and different strategies utilized to overcome this challenge.

### 1.1 Natural killer cell biology and cytotoxic mechanisms

“Naturally occurring killer lymphocytes” NK cells were first identified in 1975 with inherent cytotoxicity towards murine leukemia cells (13). NK cells are part of the innate immune system and are 5-10% of the human peripheral blood (PB) leukocytes (14, 15). They are derived from the CD34+ common lymphoid progenitors in the bone marrow and characterized by CD56 expression and absence of CD3 and TCR (16). NK cells universally express activating natural cytotoxicity triggering receptor 1 (NCR1 or NKp46) (16), and are broadly divided into two major subsets based on CD56 and CD16 (FcγRIII) expression. About 85-95% of PB NK cells are more mature CD56^dim^ CD16^high^ NK cells with high cytotoxic function, whereas the less mature CD56^bright^ CD16^-/low^ NK cells are enriched in the lymph nodes and extensively secrete cytokines (14–16). With cytokine priming (such as IL-2, IL-15), CD56^bright^ NK cells could acquire strong cytotoxic function (17).

NK cell activation and cytotoxicity are governed by the balance of signals from germline-encoded activating and inhibitory receptors. When cancer cells downregulate type I major histocompatibility complex (MHC-I) to escape cytotoxic T lymphocytes recognition, they could become more susceptible to NK cell-mediated killing due to reduced inhibition through the Killer cell Immunoglobulin-like Receptor (KIR) (15, 16). Once NK cells are activated, they can eliminate cancer cells through 1) the degranulation of lytic granules containing granzyme and perforin, 2) death receptor pathway activation where Fas Ligand (FasL) and TRAIL on NK cells bind to target cells triggering apoptosis, 3) direct CD16 signaling leading to Antibody-Dependent Cellular Cytotoxicity (ADCC) and 4) secretion of inflammatory cytokines like interferon gamma (IFNγ) and tumor necrosis factor alpha (TNF-α) and chemokines which recruit other immune cells and support antitumor response (14–16). NK cells play a crucial role in cancer immunosurveillance, helping control both primary tumors and metastasis by patrolling for transformed/abnormal cells and circulating tumor cells. Clinical studies have shown that cancer patients with reduced, exhausted, or functionally impaired NK cells correlate with worse outcomes, whereas association with tumor-infiltrating NK cells is linked to better outcomes (14).

## 2 CAR-NK and causes of “poor” CAR-NK persistence

CAR-technology utilizes synthetic receptor constructs with an extracellular antigen-binding domain, most often a single-chain variable fragment (scFv), hinge or a spacer for flexibility (H), transmembrane domain (TMD), costimulatory domain (CSD), or signaling domain (SD), which leads to immune cell activation leading to cytotoxicity upon antigen binding (1, 18). The landscape of CAR-engineered innate immune cells has now included NK, NKT, gd T cells and macrophages (3, 19, 20). The CAR-NK domains and examples are shown in **Table 1** (1, 2, 6, 7, 21–24). Early CAR-NK designs mirrored CAR-T, progressing from CD3ζ alone (first-generation) to adding one CSD (second-generation), to two CSDs (third-generation), and finally cytokine armoring (fourth-generation) to improve persistence (7, 21–25). The different generations of CAR-NK are summarized in **Table 2** (21–25).

**Table 1:** Core CAR-NK domains with examples.

CAR domain	Examples
Antigen binding domain	Cancer specific
Hinge region	IgG, CD28, CD8α
Transmembrane domain	NKG2D, 2B4, CD3, CD8, CD28, DNAM1, CD16, NKp44, NKp46
Co-stimulatory domain	4-1BB, 2B4, CD28, CD27, DAP10, DAP12, OX40, DNAM-1
Intracellular signaling domain	CD3ζ DAP10, DAP12, 2B4

**Table 2:** CAR-NK generations.

Generation	Domains	Key feature
First	ABD + H + SD	Single activation/signaling domain (example: CD3ζ)
Second	ABD + H + **CSD** + SD	Addition of a CSD to enhance proliferation, survival, activation and anti-tumor function of CAR-NK (example: 4-1BB)
Third	ABD + H + **2X** (CSD) + SD	Dual costimulatory design to further enhance CAR-NK function (example: CD28 + 4-1BB). Adds a risk of tonic signaling and exhaustion.
Fourth	ABD + H + CSD + **CAD** + SD	Autocrine cytokine production (example: IL-15) to improve persistence with or without safety switches (inducible caspase 9).
Next	ABD + H + CSD + **CAD** + SD **+** [logic-gates, adaptor-based, SynNotch, bispecific, and safety-switch etc.]	These additions can enhance safety, persistence, precision control, and signaling.

ABD, Antigen Binding Domain; H, Hinge; TMD, Transmembrane Domain; CSD, Costimulatory Domain; SD, Signaling Domain; CAD, Cytokine Armoring Domain.

**Figure 1:**
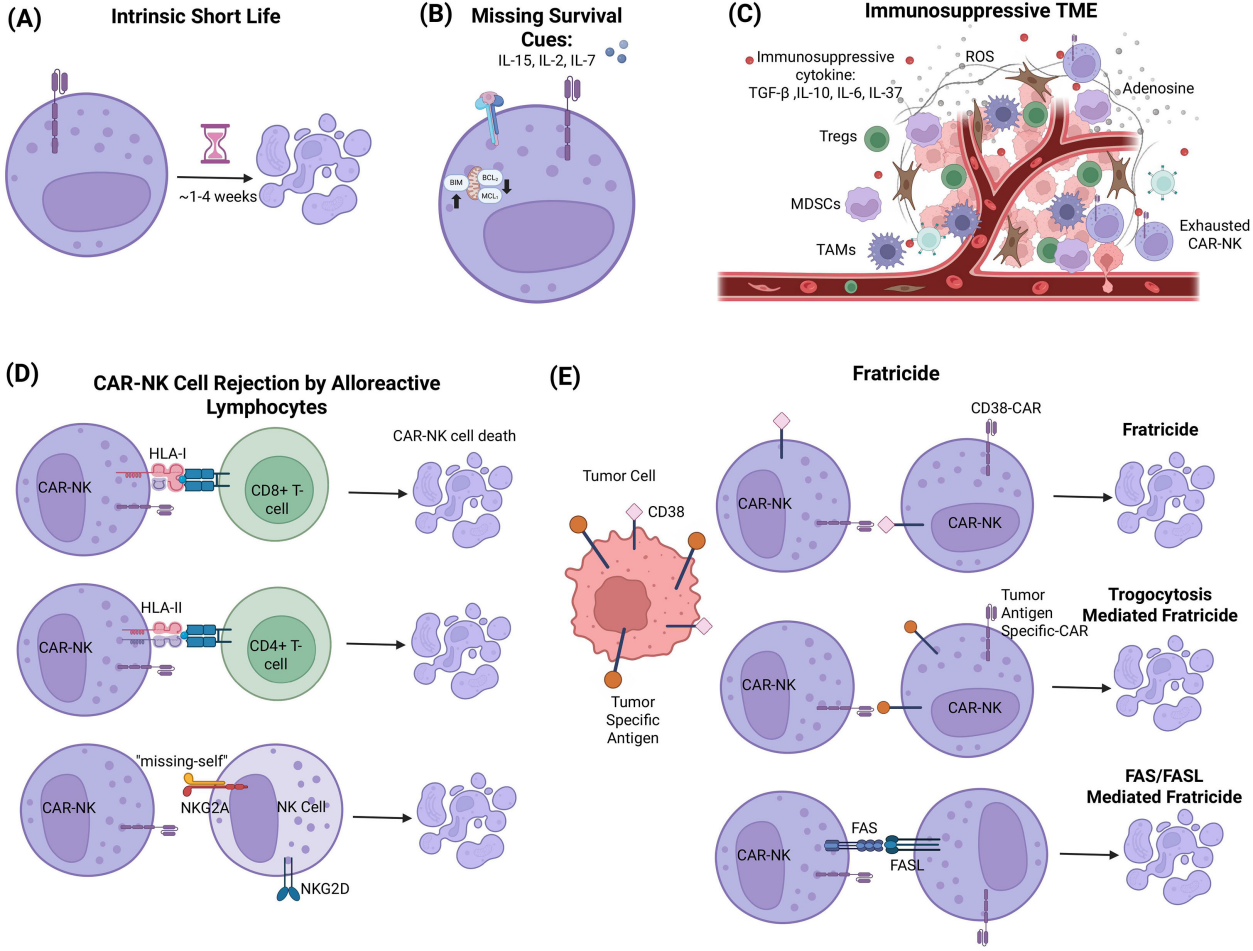
Main Causes of Poor CAR-NK Persistence. **(A)** CAR-NK cells are intrinsically short lived. **(B)** CAR-NK cells are “cytokine-addicted” and rely on survival cues (IL-7, IL-15, IL-2) from the microenvironment. **(C)** The Tumor Microenvironment (TME) is immunosupressive which can exhaust the adoptively transferred CAR-NK cells. TME can contain immunosuppresive immune cells like regulatory T-cells (Tregs), Myeloid-derived suppressor cells (MDSCs) and Tumor-Associated Macrophages (TAM); immunosupressive cytokines like TGF-β, IL-10, IL-6 and IL-37; and metabolic stressors like Reactive Oxygen Specicies (ROS) and Adenosine. **(D)** CAR-NK cells can get rejected by the host alloreactive lymphocytes. **(E)** Fratricide mediated cell death of CAR-NK cells. Created with BioRender.com.

Persistence of CAR-T and CAR-NK cells can be different (**Table 3**). T-cells, being part of the adaptive immune system, harness the power to support expansion of CAR-T cells upon antigen stimulation, and produce long-lived memory populations enabling long-term clinical remission (5, 18, 26). Unmodified CAR-NK cells are reported to be intrinsically short-lived (1–4 weeks), thereby contributing to poor *in vivo* persistence (**Figure 1A**) (9–12). Most NK cells undergo apoptosis following activation in the absence of survival cues during the rapid contraction phase (**Figure 1B**). NK cell survival is governed by common gamma chain cytokines like IL-15, IL-2, and IL-7 (27–30). It is, however, possible that the short lifespan of CAR-NK might contribute to the favorable safety profile of lower CRS, GvHD, neurotoxicity and on-target, off-tumor toxicity (1–5, 31). Therefore, it is important to note also that “unneeded” persistence may not always be advantageous. An ideal strategy should extend the persistence life span sufficient for effective tumor eradication while maintaining a favorable safety profile (18, 26).

**Table 3:** Persistence related differences between CAR-T and CAR-NK.

Feature	CAR-T cells	CAR-NK cells	Clinical implication
Typical *in vivo* persistence	~Months-years	~ 1–4 weeks (unmodified)	Persistence enhancing strategies and repeated dosing are normally used in CAR-NK
Immune lineage and memory potential	Part of adaptive immune system; can form antigen specific clones and long-lived memory	Part of innate immune system; limited intrinsic persistence; adaptive memory under specific conditions; cytokine induced memory like NK cells can be developed	Fundamental biology of the two cell types influence persistence in CAR product
Primary persistence mechanism	Antigen driven CAR-T cell expansion; long lived memory CAR-T cell formation.	Dependent on cytokine support for survival and cytokine driven memory formation	CAR-NK relies more on persistence enhancing strategies than CAR-T
Safety Profile	Prolonged persistence helps with long-term tumor control; prolong persistence can also come with adverse effects: cytopenias, hypogammaglobulinemia, neurotoxicity, secondary malignancies etc.	Limited persistence reduces immune-mediated toxicities and other adverse reactions	Shorter persistence may provide a favorable safety profile in CAR-NK

In solids tumors, tumor microenvironment (TME) is another key factor that can exacerbate the persistence of inherently short-lived NK cells (**Figure 1C**). The TME can lead to NK cell dysfunction resulting from defective proliferation, anergy, exhaustion, suppression, and plasticity (4). Chronic immunosuppressive signaling through PD-L1/PD-1, NKG2A/HLA-E, TIGIT, LAG3, TIM-3, as well as tumor-shed exosomal cargo, can downregulate NK cell activating receptors, thereby driving NK cell exhaustion and reducing persistence (7, 10, 32–34). Intrinsic checkpoints, metabolic stressors from the TME like reactive oxygen species, hypoxia, acidosis, adenosine via CD39/CD73, indoleamine 2,3-dioxygenase mediated tryptophan depletion and arginase driven arginine loss can further reduce NK persistence (7, 10, 32–34). Immunosuppressive cells like Tregs, Myeloid-derived suppressor cells (MDSCs), Tumor-Associated Macrophages (TAM), and tumor cells secrete immunosuppressive cytokines like Transforming growth factor-beta (TGF-β), IL-10, IL-6, and IL-37, further dampening NK cell persistence (7, 32–34).

Persistence of CAR-NK in solid tumors could further be defined in several ways (7, 32, 35): (i) peripheral persistence, where CAR-NK cells can be detected in the blood or a specific compartment (example: bone marrow); (ii) tumor-localized persistence, where CAR-NK cells can traffic and infiltrate into the tumor tissues; (iii) functional persistence where the CAR-NK cells still possess the ability to proliferate, mediate its antitumor functions (secrete cytokines and be cytotoxic) with repeated antigen exposure; and (iv) safe controllable persistence where the CAR-NK cells can survive and expand without severe toxicities or loss of regulatory control. In clinical studies functional persistence can also be assessed through response rates. Importantly, these different types of persistence are interconnected but not interchangeably: for example, CAR-NK cells can persist in circulation but be absent within tumor tissues or CAR-NK cells can persist in the tumor tissues but lack functional persistence due to the immunosuppressive TME. Different persistence types and their readouts are shown in **Table 4**. It is therefore important to consider the type of persistence observed under defined conditions.

**Table 4:** Different percistence readouts.

Persistence type	Definition	Persistence readout
Peripheral persistence	Continuous detection of CAR-NK cell in blood or other compartments post infusion	Flow cytometry, CAR transgene using quantitative PCR (qPCR) or Digital droplet polymerase chain reaction (ddPCR), Pharmacokinetics testing
Tumor-localized persistence	Ability of CAR-NK cells to traffic to, infiltrate, and remain within tumor tissue	Immunohistochemistry, immunofluorescence, intratumoral flow cytometry, spatial transcriptomics
Functional persistence	Continuous antitumor activity following repeated antigen exposure: cytotoxicity, serial killing capacity, cytokine secretion	Rechallenge/serial killing assays, IFNγ/TNFα production, degranulation assays, *in vivo* tumor control, clinical efficacy signals such as Overall Response Rate/Complete Response Rate or duration of response
Safe controllable persistence	Durable persistence that maintains therapeutic benefit without uncontrolled expansion or excessive toxicity	Suicide/safety switch activations, cytokine profiles, toxicity/safety assessment, clinical studies: Dose Limiting Toxicities, Cytokine Release Syndrome, Immune effector Cell-Associated Neurotoxicity Syndrome, Graft vs Host Disease

NK cells do not seem to cause GvHD and can be used safely as “off-the-shelf” allogeneic CAR-NK products (36–38). However, adoptively transferred CAR-NK cells can undergo HLA-mediated allorejection, leading to their elimination by host T-cells and NK-cells (**Figure 1D**) (6, 10). Additionally, CAR-NK cells can undergo fratricide, FasL-mediated fratricide and trogocytosis, limiting their persistence (**Figure 1E**) (6, 10, 39).

## 3 Engineering strategies to improve CAR-NK persistence

A unique feature of CAR-NK is the versatility of NK cell sources in both allogenic and autologous settings. NK cell sources include PB, Umbilical Cord Blood (UCB), NK-92 cell line, Hematopoietic Stem/Progenitor Cell (HSPC), and Induced Pluripotent Stem Cell (iPSC)/human Embryonic Stem Cells (hESC). Each source presents distinct benefits and drawbacks that shape clinical applicability. **Table 5** summarizes different NK cell sources with their advantages and limitations (1–3, 6, 22, 23). The source of NK cells could play a major role in CAR-NK *in vivo* persistence. CAR-NK cells derived from CB have higher gene expression of cell cycle and replication-related genes, compared to PB (16, 40). Additionally, CB-derived CD56+ cells can expand more than PB-derived CD56+ cells ex vivo (41). CB-CAR-NK products armored with IL-15 have been shown to persist up to a year in patients with CD19+ lymphoid malignancies (6, 38). While NK-92 cells are genetically modified and easily expandable, their oncogenic origin warrants irradiation, which drastically reduces *in vivo* persistence of the CAR-NK-92 product (6, 16, 42). These studies show how the specific intrinsic NK cell biology of each NK cell source influences persistence. CB-derived NK cells’ superior persistence can be supported by its greater proliferative capacity in comparison to PB-derived NK cells and irradiation of NK-92 cells greatly reduce their lifespan. Direct clinical comparison between CAR-NK cell sources is limited and factors like donor variability, manufacturing scalability and the differentiation status of the NK cell may influence the clinical persistence.

**Table 5:** Common CAR-NK cell sources.

Source	Source overview	Advantages	Limitations
Peripheral Blood	Mature NK cells are isolated from peripheral mononuclear cells through leukapheresis and expanded; Can be autologous or allogenic.	Easily accessible; already mature; well established isolation and expansion protocols	Low frequency in blood; variability between donors; heterogenous; expansion may cause telomere shortening, exhaustion, or reduced cytotoxic function; low viral transduction efficiency
Umbilical Cord Blood	Immature NK cells from umbilical cord blood	Higher frequency of NK cells compared to PB; high proliferative and expansion capacity; immature phenotype and reduced risk of GvHD; diverse donor availability via cord blood banks; persistence and safety proven in clinical trials.	Time consuming to expand; lower cytotoxicity; high NKG2A receptor expression (inhibitory); heterogeneous
NK-92 cell line	Immortalized cell line derived from a patient with NK cell lymphoma	Unlimited expansion; homogeneous; off-the-shelf; easy to genetically modify and stable CAR expression; NK-92 based CARs have been tested in different cancer clinical trials; high expression of activating receptors and low expression of inhibitory receptors; strong cytotoxic potential	Needs to be irradiated before infusion; irradiation lowers persistence and proliferation; the original NK-92 lack CD16 expression therefore, no ADCC unless engineered
Hematopoietic Stem/Progenitor Cell (HSPC)	NK cells differentiated and expanded from CD34^+^ hematopoietic stem cells often from cord blood	Standardized, off-the-shelf potential; unlimited expansion; preclinical/early clinical data show safety and activity in Acute Myeloid Leukemia (AML) models; easier to genetically engineer.	Low CD34+ cells and low differentiation (weeks needed, >40 days in some protocols); variability in outcomes; heterogenous; lower NK cell differentiation efficiency compared to pluripotent stem cells.
Induced Pluripotent Stem Cell (iPSC)/human Embryonic Stem Cells (hESC)	iPSCs derived NK cells are NK cells generated from reprogrammed adult somatic cells, expanded into homogeneous clonal populations. hESC derived NK cells are generated through differentiation of pluripotent cells derived from early embryos	Unlimited supply; homogeneous, standardized product; easier to genetically engineer; renewable “off-the-shelf” source.	Time-intensive production (3–5 weeks); complex differentiation protocol and need strict quality control checks; risk of malignant transformation; potential immunogenicity in iPSC derived NK cells; ethical issues with hESC-derived sources.

**Figure 2:**
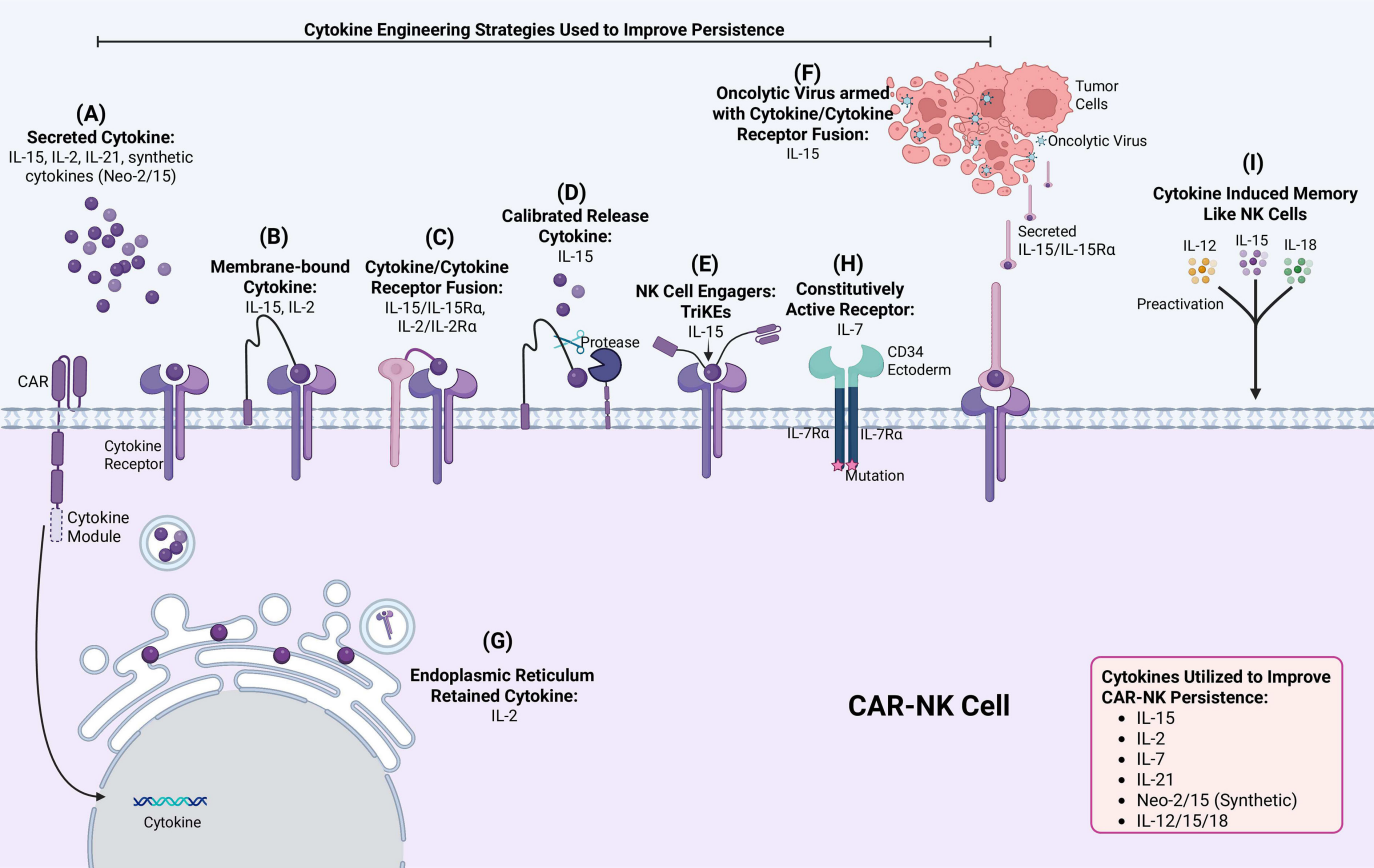
Cytokine Mediated or Cytokine Signaling Mediated Strategies to Improve CAR-NK Persistence. The most common cytokies used to improve CAR-NK persistence include, IL-15, IL-2, IL-7, IL-21, synthetic cytokines (Neo-2/15) and IL-12/15/18 for Cytokine Induced Memory Like (CIML) NK Cells. Cytokine engineering strategies include: **(A)** Secreted cytokines to the microenvironemnt for autocrine signaling (e.g. IL-15, IL-2, IL-21, Neo-2/15); **(B)** Membrane-bound cytokine engineering strategies where the cytokine is not released to the microenvironment (e.g. IL-15); **(C)** Cytokine/Cytokine-Receptor fusion stratgies (e.g. IL-15/IL-15Rα, IL-2/IL-2Rα); **(D)** Localized calibrated release of IL-15 mediated through protease cleavage of membrane bound IL-15; **(E)** NK cell engagers that could be bispecific or trispecific for localized cytokine delivery (e.g. TriKEs); **(F)** Cytokine-armored oncolytic viruses which infect cancer cells and secrete cytokine/cytokine receptor fusion which can engage with cytokine receptors on CAR-NK cells; **(G)** Endoplasmic reticulum (ER) retain cytokines where cytokine mediated signaling happens in the ER (e.g. IL-2); and **(H)** Constitutively active cytokine receptors independent of cytokine engagement (e.g. IL-7). **(I)** NK cells can be preactivated with IL-12/15/18 to make CAR-NK cells with CIML features to improve persistence. Created with BioRender.com.

Emerging evidence suggests that NK cell (subsets) can adapt to display memory-like features, improving their persistence and recall during: 1) viral infections (human/mouse cytomegalovirus); 2) priming with cytokines (IL-12, IL-15, IL-18) to produce Cytokine-induced memory-like (CIML) NK cells; and 3) hapten sensitization (31, 43, 44). Similar to T and B cells, under defined conditions, specific NK cell subset demonstrated clonal expansion and induction of long-term memory. JAK-STAT signaling pathways are highly associated with epigenetic changes induced in memory NK cells in MCMV infections. IL-12, IL-18 with IL-2 + IL-15 cytokine stimulations promoted epigenetic changes in chromatin accessibility that can be reflected in metabolisms and memory responses of the CIML NK cells (45). Such epigenetic programming may contribute to improved persistence and augmented NK cell effector functions in the immunosuppressive TME. Pre-activating mouse NK cells with IL-12/15/18 induces a mature memory-like phenotype with high CD25 and IL-2 dependence from CD4+ T cells for long-term proliferation (46). When these cells were adoptively transferred to RMA-S lymphoma-bearing mice, they robustly proliferated, expanded, cleared the tumor, and persisted for up to 3 months, unlike NK cells pretreated with IL-2/15 only. Human PB-NK cells preactivated with IL-12/15/18 have a CIML phenotype retained during proliferation (47). These cells have enhanced IFN-γ production and possess stronger recall response when restimulated with cytokines or leukemia cells after a rest period. PB-derived-CIML-NK cells have shown robust antileukemia activity and persistence in preclinical models and clinical trials of relapsed/refractory AML (48, 49). Adding a CAR to CIML-NK cell enhances anti-tumor activity by combining cytokine-driven longevity with precise antigen recognition, improving cytotoxicity and persistence (50–54). Preclinical studies have shown PB-derived CD19/NPM1-CAR-CIML-NK cells producing high levels of IFN-γ displayed efficient cytotoxicity towards leukemia and lymphoma cells (51, 52, 54). Furthermore, preclinical studies using PB-derived CAR-CIML-NK cells in xenograft models of solid tumors, like ovarian cancer and head and neck squamous cell carcinoma, have shown persistent and robust antitumor activity (50, 53). Collectively, this data shows that CIML phenotype can help CAR-NK persistence in both HM and solid tumors (**Figure 2I**).

Technological and molecular advances in gene therapy vector designs and primary NK cell culture/expansion system have made engineering of NK cells feasible. These NK cells have been engineered in multiple ways to enhance their potency against hematologic and solid malignancies (10). Here, we will mostly focus on strategies that promote NK cell persistence *in vivo*. We have summarized key selected studies, based on Broad Strategy, CAR NK product, Disease Context, Persistence Type, Persistence Readout/Efficacy Signal/Safety, to establish a clearer framework for evaluating CAR-NK persistence enhancing strategies (**Table 6**).

**Table 6:** Persistence enhancing strategies in CAR-NK cell therapy.

Broad strategy	CAR-NK product	Disease context and evidence level	Persistence type	Persistence readout, efficacy signal and safety	Reference
Cytokine Armoring (IL-15)	NK Cell Source: CB; • Engineering Modality: iC9 suicide gene + CD28/CD3ζ CD19-CAR + secreted IL-15	Hematologic Malignancy: CD19+ B-cell malignancies; Evidence Level: Phase I/II	Peripheral Persistence; Functional Persistence; Safe Controllable Persistence	CAR-NK cells detected up to 1 year in peripheral blood; ORR 48.6% (day 30 and day 100); 1-year OS 68%; 1-year PFS 32%; iCasp9 safety switch; No neurotoxicity; No GvHD; 1 Grade 1 CRS event	(37, 38, 64) (NCT03056339)
Cytokine Armoring (IL-15)	NK Cell Source: CB; Engineering Modality: CD19-CAR (4-1BB/CD3ζ) + secreted IL-15	Hematologic Malignancy: R/R large B-cell lymphoma; Evidence Level: Phase I	Peripheral Persistence; Functional Persistence; Safe Controllable Persistence	Peak at 4–7 days post-infusion; CAR copies detected in peripheral blood up to 15 months post-infusion; No CAR copies detected in non-responders by 2 weeks; ORR 62.5% at day 30; CR in 4 patients (50%); Median PFS 9.5 months; Median OS not reached; No dedicated safety switch; No DLTs, CRS, neurotoxicity, or GvHD	(67) (NCT05472558)
Cytokine Armoring (IL-15)	NK Cell Source: PB; Engineering Modality: CD123-CAR (2B4. CD3ζ) + secreted IL-15	Hematologic Malignancy: AML; Evidence Level: Preclinical (*in vitro* + xenograft models)	Peripheral Persistence	Robust expansion up to 31 days post-infusion; Potent anti-AML activity in MOLM13 and MV-4–11 xenograft models. No dedicated safety switch; Lethal toxicity in MV-4–11 xenograft model	(69)
Cytokine Armoring (IL-15)	NK Cell Source: CB; Engineering Modality: CD33-CAR (4-1BB/CD3ζ) + secreted IL-15	Hematologic Malignancy: R/R AML; Evidence Level: Phase I	Peripheral Persistence; Functional Persistence; Safe Controllable Persistence	CAR-NK detected 7 days post-infusion; Peak expansion observed right after each infusion; MRD-negative CR in 6/10 patients by day 28; No dedicated safety switch; Grade 2 CRS in 1 patient; No ICANS or GvHD	(12) (NCT05008575)
Cytokine Armoring (IL-15)	NK Cell Source: PB; Engineering Modality: NKG2D-CAR (OX40/CD3ζ) + mbIL-15 (NKX101)	Hematologic Malignancy: R/R AML; Evidence Level: Phase I	Peripheral Persistence; Functional Persistence; Safe Controllable Persistence	NKX101 detected up to 3 weeks in peripheral blood CR/CRi in 4/6 patients; CR in 3/6; 3/6 MRD negativity by flow; Full efficacy data pending; No dedicated safety switch; no CRS, ICANS, or GvHD	(71, 72) (NCT04623944)
Cytokine Armoring (IL-15)	NK Cell Source: PB; Engineering Modality: CD19-CAR (OX40/CD3ζ) + mbIL-15 (NKX019)	Hematologic Malignancy: R/R B-cell lymphoma; Evidence Level: Phase I	Peripheral Persistence; Functional Persistence; Safe Controllable Persistence	*In vivo* half-life up to 28 days without systemic IL-2 support; ORR 80%; CR 70% at highest dose level (1–1.5×10^9^ cells); No dedicated safety switch; no DLTs, ICANS, GvHD, or Grade >3 CRS; Minimal cytokine elevations	(73, 74) (NCT05020678)
Cytokine Armoring (IL-15)	NK Cell Source: iPSC; Engineering Modality: CD19-CAR (NKG2D/2B4/CD3ζ) + hnCD16 + IL-15/IL-15Rα fusion (FT596)	Hematologic Malignancy: R/R B-cell lymphoma; Evidence Level: Phase I	Peripheral Persistence; Functional Persistence; Safe Controllable Persistence	FT596 detectable in peripheral blood up to 15 days; ORR 54%; CR 37%; Durable responses observed; No dedicated safety switch; No ICANS, GvHD, Grade >3 CRS, or treatment-related deaths	(36) (NCT04245722)
Cytokine Armoring (IL-7)	NK Cell Source: PB; Engineering Modality: Constitutively active IL-7Rα (C7R) + GD2-CAR or CD19-CAR (4-1BB/CD3ζ)	Hematologic Malignancy: B-Cell Leukemia; Solid tumor: neuroblastoma; Evidence Level: Preclinical (*in vitro* + xenograft)	Peripheral Persistence	Sustained CAR-NK bioluminescent signal and survival *in vivo* despite cytokine withdrawal; Reduced cytotoxicity; Reduced long-term tumor control; No dedicated safety switch; No formal safety assessment	(86)
Cytokine Armoring (IL-21)	NK Cell Source: PB; Engineering Modality: CD19-CAR (4-1BB/CD3ζ) + Secreted IL-21	Hematologic Malignancy: B-cell lymphoma; Evidence Level: Preclinical (*in vitro* + xenograft)	Peripheral Persistence; Functional Persistence	Increased CAR-NK survival; Enhanced serial killing after repeated tumor challenge; Higher CAR-NK in spleen, liver, and bone marrow at endpoint; Superior Raji lymphoma control and prolonged survival; No dedicated safety switch; No formal safety assessment beyond body-weight monitoring	(87)
Cytokine-Induced Memory-like	NK Cell Source: PB; Engineering Modality: CIML induction (IL-12/IL-15/IL-18) + MSLN-CAR (4-1BB/CD3ζ)	Solid Cancer: Ovarian cancer; Evidence Level: Preclinical (*in vitro* + xenograft)	Functional Persistence	Sustained cytotoxicity; IFN-γ production, and degranulation during prolonged tumor challenge; Maintained antitumor activity after exposure to ovarian cancer ascites; Enhanced tumor control; Reduced metastasis; Improved survival; Reduced ascites burden in ovarian cancer xenografts; No dedicated safety switch; No formal safety assessment	(50)
CAR Design Optimization	NK Cell Source: CB; Engineering Modality: CD70-targeting CD27-CAR (CD28/CD3ζ) + secreted IL-15	Hematologic Malignancy: Lymphoma, AML; Solid Cancer: breast cancer; ovarian cancer; Evidence Level: Preclinical (*in vitro* + xenograft)	Peripheral Persistence; Tumor-Localized Persistence; Functional Persistence	Higher circulating NK-cell counts at days 10–20; increased tumor/lung infiltration; sustained cytotoxicity after repeated tumor rechallenge; Superior tumor control and prolonged survival across hematologic and solid tumor xenograft models; No dedicated safety switch; No cytotoxicity against normal hematopoietic or immune cells reported	(57)
CAR Design Optimization	NK Cell Source: UCB CD34^+^ HSC-derived NK; Engineering Modality: CD19-CAR (CD28/CD3ζ)	Hematologic Malignancy: B-cell leukemia/lymphoma; Evidence Level: Preclinical (*in vitro* + xenograft)	Functional Persistence	Enhanced proliferation and survival during repetitive tumor challenge; Sustained cytotoxicity across repeated antigen exposure; Improved tumor control and prolonged survival compared with alternative CAR designs; No dedicated safety switch; No formal toxicity assessment	(59)
Intrinsic Checkpoint Disruption	NK Cell Source: CB; Engineering Modality: CISH KO + iC9-CD19-CAR(CD28/CD3ζ) + secreted IL-15	Hematologic Malignancy: Lymphoma; Evidence Level: Preclinical (*in vitro* + xenograft)	Peripheral Persistence; Functional Persistence; Safe Controllable Persistence	Improved peripheral CAR-NK persistence up to 7 weeks post-infusion; Enhanced cytotoxicity versus either modification alone; Eradication of lymphoma xenografts; prolonged survival; Superior tumor control versus either modification alone; iCasp9 safety switch; No increased systemic cytokines, organ damage, uncontrolled expansion, or autonomous growth	(99)
Intrinsic Checkpoint Disruption	NK Cell Source: CB; Engineering Modality: CREM KO + CAR-NK (CD28/CD3ζ) + secreted IL-15	Hematologic Malignancy: Lymphoma; Solid Cancer: breast, pancreatic cancer; Evidence Level: Preclinical (*in vitro* + xenograft)	Peripheral Persistence; Tumor-Localized Persistence; Functional Persistence; Safe Controllable Persistence	Higher circulating CAR-NK-cell counts at days 10 and 20; Enhanced tumor infiltration; Preserved cytotoxicity following repeated antigen challenge; Superior tumor control across multiple models; Prolonged survival in lymphoma and breast cancer xenografts; No dedicated safety switch; No weight loss, organ pathology, hematologic abnormalities, or serum chemistry changes	(66)
Intrinsic Checkpoint Disruption	NK Cell Source: CB; Engineering Modality: Dual (ARIH2/CCNC) KO + CD27/TROP2- CAR(2B4/CD3ζ) + secreted IL-15	Hematologic Malignancy: Multiple Myeloma; Solid Cancer: Pancreatic cancer; Evidence Level: Preclinical (*in vitro* + xenograft)	Peripheral Persistence; Tumor-Localized Persistence; Functional Persistence	Higher circulating CAR-NK-cell counts; Enhanced tumor infiltration post-infusion; Superior tumor control; Prolonged survival in xenograft models; No dedicated safety switch; No formal toxicity assessment; No body-weight changes or cytokine-independent growth	(101)
Intrinsic Checkpoint Disruption	NK Cell Source: PB; Engineering Modality: Dual KLRC1/FAS KO + CD19-CAR (CD28/CD3ζ)	Hematologic Malignancy: B-ALL, lymphoma; Evidence Level: Preclinical (*in vitro* + xenograft)	Peripheral Persistence; Functional Persistence	Higher circulating CAR-NK-cell counts *in vivo* (day 7); enhanced serial-killing capacity following repetitive stimulation; Improved leukemia control; prolonged survival in a donor-dependent manner; Enhanced target-cell clearance during repetitive challenge; No dedicated safety switch; No formal toxicity assessment	(105)
Extrinsic Checkpoint Disruption	NK Cell Source: iPSC; Engineering Modality: TGFBR2 KO + anti-GPC3/AFP CAR (NKG2D/2B4/CD3ζ)	Solid Cancer: Hepatocellular carcinoma; Evidence Level: Preclinical (*in vitro* + xenograft)	Peripheral Persistence; Functional Persistence	Increased peripheral NK-cell persistence (days 7-14); Sustained cytotoxicity and cytokine production; Superior tumor control; Prolonged survival in xenograft models; No dedicated safety switch; No formal toxicity assessment	(106)
Extrinsic Checkpoint Disruption	NK Cell Source: PB; Engineering Modality: NKG2D-CAR (4-1BB/CD3ζ) + mbIL-15+ anti-PD-1 antibody	Solid Cancer: Metastatic colorectal cancer; Evidence Level: Phase I exploratory	Peripheral Persistence	Peripheral CAR transgene detection up to 2 weeks post-infusion; Higher peak CAR copies with PD-1 blockade; Preliminary clinical efficacy; Modest clinical activity; SD in 1 patient; Prolonged OS (>700 days) in 2 patients receiving PD-1 blockade; Small cohort (n=3 per arm); No dedicated safety switch; Favorable clinical safety profile with no Grade ≥3 CRS, neurotoxicity, GvHD, or treatment-related death	(110)
Extrinsic Checkpoint Disruption	NK Cell Source: NK-92; Engineering Modality: TGFβRII/IL-21R switch receptor + NKG2D-CAR (4-1BB/CD3ζ)	Solid Cancer: Gastric cancer; Evidence Level: Preclinical (*in vitro* + xenograft)	Tumor-Localized Persistence; Functional Persistence	Increased proliferation; Reduced apoptosis; Higher intratumoral NK-cell infiltration; Superior tumor control; Reduced tumor burden; Prolonged survival; No major toxicity; Stable body weight; No histopathologic abnormalities in major organs	(111)
Gain-of-Function Engineering	NK Cell Source: CB and PB; Engineering Modality: OR7A10 GPCR overexpression + HER2-CAR (CD28-4-1BB/CD3ζ) + secreted IL-15	Solid Cancer: Colon, breast, ovarian cancer; Evidence Level: Preclinical (*in vitro* + xenograft)	Peripheral Persistence; Tumor-Localized Persistence; Functional Persistence	Increased proliferation; Repeated-challenge killing; Reduced exhaustion; Enhanced TME resistance; Increased NK-cell accumulation in tumor, blood, and spleen after transfer; Strong *in vivo* efficacy across multiple solid tumor models; 100% CR (5/5 mice) with long-term tumor control and prolonged survival in an orthotopic breast cancer model; No dedicated safety switch; Enhanced persistence without detectable cytokine toxicity, organ pathology, genomic instability, or transformation	(116)
Multiplex Checkpoint Editing	NK Cell Source: PB; Engineering Modality: Triple KO (CISH, PDCD1, TIGIT) + CD19-CAR+ secreted IL-15	Hematologic Malignancy: Lymphoma; Evidence Level: Preclinical (*in vitro* + xenograft)	Peripheral Persistence; Functional Persistence	Increased NK-cell expansion/persistence in peripheral blood, bone marrow, and spleen at endpoint; Sustained tumor control *in vivo*; Improved tumor control and survival versus; No dedicated safety switch; Excessive CAR-NK expansion leading to systemic toxicity and rapid weight loss in some mice	(118)
Alloevasion	NK Cell Source: iPSC; Engineering Modality: B2M KO + CIITA KO + HLA-E KI + EGFRKI + CD19-CAR+ secreted IL-15 (CNTY-101)	Hematologic Malignancy: R/R CD19+ B-cell malignancies; Evidence Level: Phase I	Peripheral Persistence; Functional Persistence; Safe Controllable Persistence	Detectable in blood up to 28 days post-infusion; Persistence maintained without lymphodepletion during repeated dosing; Clinical responses observed; ORR/CRR 25%/25% (100×10^6^ cells); ORR/CRR 67%/33% (300×10^6^ cells); EGFR KI enables cetuximab-mediated elimination switch; No DLTs, GvHD, or ICANS; 1 Grade 1 CRS; 1 Grade 2 CRS	(126, 127) (EliPSE-1; NCT05336409)
Alloevasion	NK Cell Source: iPSC; Engineering Modality: ADR + CD38 KO + hnCD16 + CD19-CAR+ IL-15/IL-15 RF (FT522)	Hematologic Malignancy: R/R B-cell lymphoma; Evidence Level: Phase I	Peripheral Persistence; Functional Persistence; Safe Controllable Persistence	CAR-NK detectable post-infusion with and without lymphodepletion (day 15); Delayed recovery of 4-1BB^+^ T cells consistent with ADR activity *in vivo*; CR in 2/3 patients; No dedicated safety switch; No CRS, ICANS, GvHD, or DLTs	(129) (NCT05950334)
Fratricide Evasion & Alloevasion	NK Cell Source: iPSC; Engineering Modality: CD70 KO-CD70-CAR (4-1BB/CD3ζ) + hnCD16+ IL-15/IL-15Rα fusion (70CAR-iNK)	Hematologic Malignancy: CD70+ lymphoma; Solid Cancer: RCC; Evidence Level: Preclinical (*in vitro* + xenograft)	Peripheral Persistence; Functional Persistence	70CAR-iNK cells detected 28 days post-infusion in blood; Resistance to alloreactive T-cell rejection; Sustained cytotoxicity during repeated tumor rechallenge assays; Improved lymphoma tumor control and survival in xenografts; Sustained killing of lymphoma and RCC targets *in vitro*; No dedicated safety switch; No formal toxicity assessment	(131)
Fratricide Evasion	NK Cell Source: CB; Engineering Modality: iC9 + Tumor-targeting CAR + KIR2DL1-based inhibitory CAR (ITIM) +secreted IL-15	Hematologic Malignancy: B-cell lymphoma, AML; Solid Cancer: Ovarian; Evidence Level: Preclinical (*in vitro* + xenograft)	Peripheral Persistence; Tumor-Localized Persistence; Functional Persistence	More CAR-NK cells detected in blood up to day 30; Reduced TROG^+^-mediated fratricide and hyporesponsiveness; Improved tumor infiltration Improved tumor control; Reduced tumor burden; Prolonged survival in hematologic malignancy and ovarian cancer xenograft models iCasp9 safety switch; No formal toxicity assessment	(136)
FasL/FAS Circuit Disruption	NK Cell Source: CB; Engineering Modality: CD19- CAR (1928ζ) + dominant-negative FAS receptor (ΔFAS)	Hematologic Malignancy: B-cell lymphoma, B-ALL; Evidence Level: Preclinical (*in vitro* + xenograft)	Peripheral Persistence; Functional Persistence	Bone marrow enrichment *in vivo*; Accumulation during serial *in vitro* tumor restimulation assays; Enhanced xenograft survival at low E:T ratios; FASLG dispensable for CAR-NK cytotoxicity; No dedicated safety switch; no toxicities reported	(39)

ADR, Alloimmune Defense Receptor; AML, Acute myeloid leukemia; B, ALL, B, cell acute lymphoblastic leukemia; B2M, Beta, 2, microglobulin; CB, Cord blood; CIITA, Class II MHC transactivator; CIML, Cytokine, induced memory, like; CR, Complete response; CRi, Complete response with incomplete hematologic recovery; CRR, Complete response rate; CRS, Cytokine release syndrome; DLT, Dose, limiting toxicity; E:T, Effector, to, target ratio; GvHD, Graft, versus, host disease; HCC, Hepatocellular carcinoma; hnCD16, High, affinity non, cleavable CD16; HSC, Hematopoietic stem cell; iCasp9/iC9, Inducible caspase, 9; ICANS, Immune effector cell, associated neurotoxicity syndrome; iPSC, Induced pluripotent stem cell; ITIM, Immunoreceptor tyrosine, based inhibitory motif; KI, Knock, in; KO, Knockout; mbIL, 15, Membrane, bound IL, 15; MRD, Minimal residual disease; NK, Natural killer; ORR, Overall response rate; ORR/CRR, Overall response rate/Complete response rate; OS, Overall survival; PB, Peripheral blood; PFS, Progression, free survival; R/R, Relapsed/refractory; RCC, Renal cell carcinoma; RF, Receptor fusion; SD, Stable disease; TME, Tumor microenvironment; TROG^+^, Trogocytosis, positive; UCB, Umbilical cord blood.

### 3.1 CAR design

CAR design can influence CAR-NK persistence. NK-specific CSDs like DNAM1 or 2B4 have been shown to yield CAR-NK-92 cells with higher proliferation, lower apoptosis, and survival advantage, unlike T-cell-derived domains like CD28 or first-generation CARs in hepatocellular carcinoma (HCC) cell lines (55). Comparing seven CB-derived CD19-CAR-NK designs, the utilization of OX40 as a CSD improved CAR-NK persistence *in vivo* in a CD19^+^ B-ALL model (56). Utilizing CB-derived IL-15-armored CD70-CAR-NK, CD28, 4-1BB, DAP10, or DAP12 domains were tested and found CD28 provided superior persistence through AKT activation downstream of LCK-CD3ζ-ZAP70 axis (57, 58). This led to phase I/II clinical trials using CD28-based CAR-NK therapies for CD70^+^ HM and solid tumors (NCT05703854, NCT05092451) (57). Another CB-CAR-NK study validated CD28 as a key CSD outperforming 4-1BB or CD3ζ alone CAR designs with improved persistence and long-term cytotoxicity in a B-ALL model (59). The CD28-mediated improvement in persistence may be NK source dependent as CD28-CD3ζ displayed better tumor control capacities than 4-1BB-CD3ζ, but had similar persistence in CAR-NK-92 in HCC model (60). CD28-CD3ζ had superior cytotoxicity through MAP3K8/ERK activation-mediated tumor control (60). In multiple solid tumor models, synapse-tuned PB-CAR-NK improved tumor control, activation, and putatively improved long-term persistence (58, 61). Synapse-tuning incorporates a PDZ-binding motif into the CAR endodomain, which recruits scaffold proteins like Scribble, strengthening signaling (61).

CD28 outperforms several other CSDs in multiple CAR-NK models, however, these effects are not consistent across different NK cell sources. Therefore, optimal CAR CSD is dependent on the NK cell source and disease context and findings from one model might not be generalizable to all models. Additionally, CAR designs need to be optimized to prevent excessive tonic signaling as this can accelerate NK cell dysfunction and exhaustion. Therefore, balanced activation signals are important not only to enhance pro-survival pathways but to minimize activation induced stress and exhaustion that can limit NK cell persistence.

### 3.2 Cytokine and cytokine signaling mediated strategies

Cytokine engineering remains the most used method to improve CAR-NK persistence. Prior to CAR transduction, NK cells are primed and expanded *in vitro* for optimal expansion, persistence, and antitumor activity. This is primarily done by using cytokines (IL-2, IL-15, IL-21), feeder cell systems like engineered K562 cells with stimulatory ligands and membrane-bound cytokines, and antibody-based activation strategies (anti-CD2 and anti-NKp46) (62, 63). When cytokine-addicted CAR-NK cells are infused into patients, they could “struggle” to persist given the lack of constant cytokine-mediated support. As systemic administration of cytokines poses a safety risk, cytokine-armored CAR-NK cells have been developed to improve persistence (7).

#### 3.2.1 Interleukin-15

IL-15 is the most widely explored cytokine armoring strategy to improve CAR-NK persistence. Rezvani lab engineered IL-15-armored CB-derived CD19-CAR-NK with an inducible caspase-9-based suicide gene as a safety switch (64). Armored CAR-NK cells secreted IL-15 in an antigen-driven autocrine loop sustaining their survival and persistence without NK cell exhaustion or anergy, eliminating leukemia cells and persisting up to 68 days in xenograft lymphoma models. Importantly, administration of IL-15-armored CAR-NK cells in patients with relapsed or refractory CD19+ lymphoid malignancies enrolled for phase 1/2 trial (NCT03056339) led to complete remission in 7/11 patients and persisted up to a year post-infusion (37, 38). Secreted IL-15 (**Figure 2A**) in CAR-NK has been explored in both preclinical and clinical settings to improve persistence in both HM and solid tumors (37, 57, 65–68). While IL-15 armoring is generally safe in iPSC- and UCB-derived CAR-NKs, constitutive IL-15 secretion by PB-CAR-NK cells improved persistence but induced lethal systemic inflammation in Acute Myeloid Leukemia (AML), highlighting the importance of controlled, source-specific IL-15 secretion (69).

Alternative strategies have been developed to activate IL-15 signaling without cytokine secretion. In membrane-bound IL-15 (mbIL-15) NK cells, autocrine IL-15 signaling activates anti-apoptotic pathways, supporting NK cell survival and expansion, is a safer strategy without cytokine secretion (70)(**Figure 2B**). This concept has been translated into CAR-NK therapy. NKX101 is an allogeneic mbIL-15-expressing PB-CAR-NK product currently being evaluated in clinical trials for AML (NCT04623944) (71, 72). Preliminary clinical results report NKX101 persistence up to three weeks in AML patients with early responses without reported CRS or neurotoxicity. Additionally, NKX019 an allogenic CD19-NK cell product with mbIL-15 to improve persistence is being tested in clinical trials for B-cell cancers (NCT05020678) and immune-mediated diseases like Systemic Lupus Erythematosus (NCT06733935) (73, 74). Independent of systemic IL-2 support, the addition of mbIL-15 improved persistence up to 28 days (*in vivo* half-life) (74). Furthermore, membrane-bound IL-15/IL-15Rα fusions (**Figure 2C**) have been incorporated into iPSC-CAR-NK cells in both preclinical and clinical studies (NCT04245722, NCT05182073 and NCT05950334) to enhance survival, expansion, and *in vivo* persistence independent of exogenous cytokine in HM (36, 75–77). A novel strategy enabling precise, calibrated release of IL-15 mediated through protease cleavage of mbIL-15 in PB-CAR-NK is now being explored in AML (**Figure 2D**) (6).

NK cell engagers are utilized to deliver IL-15 signaling (**Figure 2E**). A modified human IL-15 crosslinker embedded within trispecific killer engagers (TriKEs) improved patient NK-cell persistence in AML xenograft models by providing localized IL-15 survival signaling directly to NK cells promoting persistence (78). A second-generation TriKE has been generated with humanized CD16 binding domain that improved IL-15 delivery and has been translated into phase I clinical trials (77, 79) (NCT06594445). Oncolytic viruses have also been utilized to deliver IL-15. PB-derived EGFR-CAR-NK cells used synergistically with IL-15/IL-15Rα armed herpes simplex virus and showed synergistic antitumor activity and improved CAR-NK persistence without systemic toxicity in glioblastoma (**Figure 2F**) (80). Overall, immense evidence shows that cytokine armoring CAR-NK cells with IL-15 improves persistence in AML and solid tumors (6, 81).

IL-15 seems to promote overall persistence of CAR-NK cells, despite some variation in the results. For example, engineered IL-15-armored CB-derived CD19-CAR-NK (NCT03056339), for CD19+ B cell malignancies, persisted up to a year; whereas CB-derived CD33-CAR-NK (NCT05008575) for relapsed/refractory AML had poor persistence (~7 days) (12, 37). Although both CB-derived CAR-NK products used similar persistence enhancing strategy of secreted IL-15, CD19-CAR-NK persisted longer than CD33-CAR-NK cells. This difference could be stemming from factors like the quality of CB used, target antigen, the costimulatory domain of the CAR used and the immunosuppressive TME associated with AML. Poor persistence (~15 days) was also observed in FT596, an iPSC-derived CD19-CAR-NK with an IL-15/IL-15Rα, in B-Cell Lymphoma (BCL) (36). IL-15 directly improves NK cell survival and metabolic fitness and stands as the most clinically validating strategy for improving CAR-NK persistence. However, as seen by the variability in persistence data, IL-15 signaling alone cannot improve persistence as the persistence of a CAR-NK product is also influenced by NK cell source, disease context, CAR design and the TME. Depending on the NK cell source, IL-15 can also pose systemic toxicities, therefore incorporation of a suicide switch would be beneficial in clinical studies. Additionally, more controlled IL-15 signaling through mbIL-15 or IL-15 receptor fusions would offer a more favorable safety profile than constitutively secreted IL-15.

#### 3.2.2 Interleukin-2

In the FT516 iPSC-NK with a high affinity, non-cleavable CD16, clinical trial for BCL, low dose of exogenous IL-2 is administered with NK cells to improve persistence. However, the persistence of FT516 was 3–8 days (82). As systemic treatments can cause adverse side-effects, clinical study targeting locally advanced or metastatic solid cancers (NCT04050709) utilizes autocrine IL-2 signaling (by retaining IL-2 in the endoplasmic reticulum) in the PD-L1-targeted CAR-NK-92 with high-affinity CD16 cells (**Figure 2G**) (6, 83). IL-2/IL-2Rα fusion receptors (**Figure 2C**) are also used for *in vitro* preclinical studies to improve overall CAR-NK-92 survival by limiting apoptosis while enhancing proliferation (84). CAR-NK-92 cells armored with synthetic neoleukin-2/15 (**Figure 2A**), an IL-2Rβγ agonist, activate STAT5-Akt-c-Myc-NRF1 signaling to improve metabolic fitness, reduce exhaustion, and prolong *in vivo* persistence with superior antitumor effects in ovarian and pancreatic cancer models (85). Collectively, ER-retained, cytokine/receptor fusion, or the neo-2/15 mediated IL-2 cascade can improve CAR-NK cell persistence by enhancing survival.

Even though IL-2 can improve survival and proliferation in CAR-NK cells, the effects seem to be less robust than IL-15. IL-2 has broader biological effects like regulatory T cell activation and systemic toxicities which could explain the lower persistence capabilities when compared to IL-15. To minimize systemic toxicities and off-target immune activation, the field has shifted towards making more localized IL-2 signaling systems like retaining IL-2 to the ER. However, if these would improve clinical efficacy and persistence is yet to be determined.

#### 3.2.3 Interleukin-7

Previous reports shown that IL-7 promoted survival of CD56^bright^ NK cells through increased Bcl2 expression and decreased apoptosis (29). Recently, constitutively activated IL-7 receptor signaling leading to downstreamSTAT5 activation has been explored in preclinical PB-CAR-NK study in both CD19+ leukemia and neuroblastoma models (**Figure 2H**) (86). Although this improved CAR-NK persistence, chronic signaling led to a stress-associated transcriptional profile with reduced cytotoxicity. This is a clear example of how improved persistence does not necessarily translate to superior cytotoxicity and highlights the broader challenge of balancing persistence with CAR-NK antitumor functionality.

#### 3.2.4 Interleukin-21

Recent preclinical studies show that IL-21 armoring is superior to IL-15 (87–89). In glioblastoma and BCL models, IL-21-engineered NK cells (CB-derived-NK and PB-derived CD19-CAR-NK cells) demonstrated improved antitumor activity, persistence, and cytotoxicity compared to IL-15 counterparts (87, 88). Mechanistically, IL-21 activated STAT1/3, inducing CEBPD, enhancing chromatin accessibility at genes regulating metabolism, survival, cytotoxicity, improved mitochondrial fitness, and supported long-term persistence (88). IL-21-engineered NKG2D-CAR-NK-92 cells in lung cancer models showed activated AKT signaling, increased proliferation, and cytotoxicity with reduced apoptosis and TIM-3 expression (89). Collectively, these studies highlight IL-21 as a promising strategy to improve CAR-NK persistence (**Figure 2A**). Interestingly IL-21 induced long-term metabolic programming of CAR-NK cells outperformed IL-15 counterparts, however this was done in preclinical studies. Clinical studies comparing the two cytokines are lacking and if IL-21 is superior to IL-15 in the clinic is yet to be determined.

### 3.3 Checkpoint disruption, signaling modulation and metabolic reprogramming

**Figure 3:**
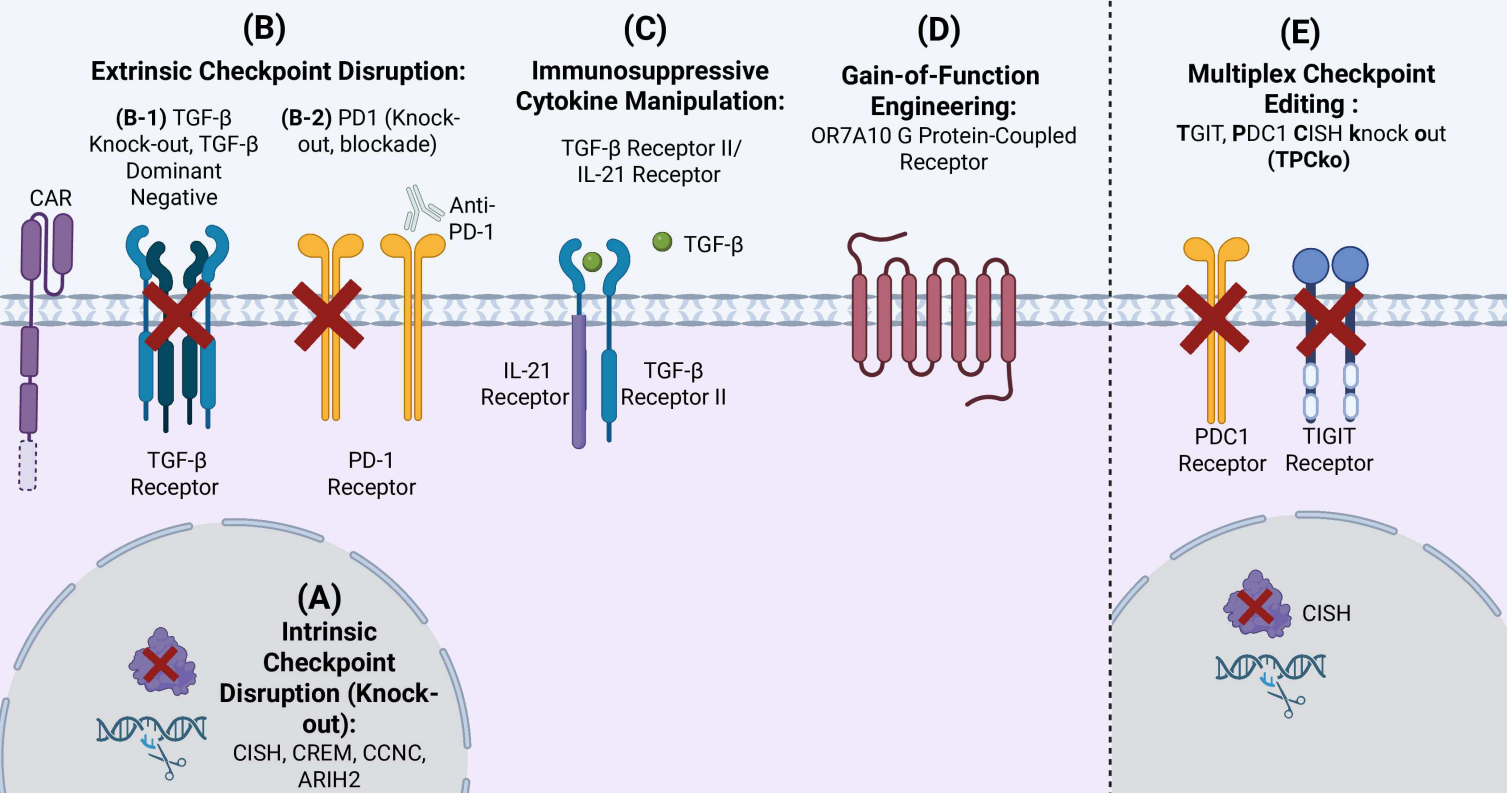
Checkpoint Disruption Strategies Used to Improve CAR-NK Persistence. **(A)** Intrisic checkpoints can be knocked out to improve CAR-NK persistence (e.g. CISH, CREM, CCNC, AIH2, MED12). **(B)** Extrinsic checkpoints can be knocked out or inhibited throgugh antibodies to improve CAR-NK persistence (e.g. (B-1)TGF-β receptor can be knocked out or a dominant negative form of the receptor can be used, (B-2)PD-1 receptor can be knocked out or blocked with a anti-PD-1 antibody). **(C)** Immunosupressive cytokines in the microenvironment can also be manipulated into a survival signal, thus improving persistence through the receptor manipulation, where TGF-β receptor is modified to TGF-βRII/IL-21R. **(D)** Gain-of-Function engineering strategies can improve CAR-NK persistence **(E)** Finally, multiplex-checkpoint editing enables intrinsic and extrinsic checkpoints to be knocked out at the same time to improve CAR-NK persistence. Created with BioRender.com.

Targeting intrinsic (**Figure 3A**) and extrinsic checkpoints (**Figures 3B, C**) with metabolic reprogramming are strategies to prolong CAR-NK survival by relieving exhaustion and sustaining cytokine signaling.

#### 3.3.1 Intrinsic checkpoint disruption and metabolic rewiring

NK-cell development and functional maturation are regulated by miRNA (90) or transcription factors (91), such as Id2, Nfil3, Foxo1, T-bet, Eomes (91–93). Deeper mechanistic studies reveal further underlying signaling axes involved in these biological processes. For examples, miR155HG is shown to bind to miR-6756 to relieve its repression of JAK3, and therefore promoted a JAK-STAT signaling loop in NK (94). Recent work by Huang et al., reported a novel GFI1-FOXO1 axis (95) that regulates the EOMES-T-BET balance critical for NK cell development. These studies, collectively, will lead to identification of novel targets for improving NK-cell fitness in immunotherapy (96).

Removing intracellular brakes that limit IL-15 responsiveness and metabolic fitness, which improves persistence. Cytokine-inducible SH2-containing (CISH) is an important intracellular checkpoint in NK cells that negatively regulates IL-15 signaling. CISH inhibits JAK1 activity and targets it for proteasomal degradation. CISH deletion makes NK cells hypersensitive to IL-15, increasing JAK-STAT signaling, enhancing proliferation, survival, and cytotoxicity towards melanoma, breast and prostate tumors (97). CISH-knockout (KO) iPSC-NK cells show increased metabolic fitness, increased sensitivity to IL-15, improved antitumor activity, and enhanced persistence through mTOR signaling in AML (98). This has been translated to CB-CAR-NK cells combining CISH deletion with IL-15 armoring, which enhances aerobic glycolysis via activation of the Akt/mTORC1-MYC pathway, resulting in greater glycolytic metabolism, persistence, and antitumor efficacy in lymphoma models (99).

Another intrinsic checkpoint in NK cells is the Transcription Factor (TF) cyclic AMP response element modulator (CREM), an important intracellular checkpoint (66, 100). CREM induction coincides with CAR-NK cell activation and dysfunction after adoptive transfer during peak effector function. CREM is a transcription regulator and epigenetically reprograms CB-CAR-NK cells. CREM is induced downstream of IL-15 signaling and CAR-CD3ζ through the PKA-CREB pathway. CREM deletion improved resistance to tumor-induced immunosuppression after rechallenging, enhanced effector function, and improved persistence in lymphoma models. CREM-KO CAR-NK cells had improved persistence compared to WT CAR-NK cells in lymphoma, breast and pancreatic cancer models. This improved persistence could be explained by the closed ETS motifs and enriched AP-1 motifs in CREM-KO CAR-NK cells, which are epigenetic signatures resembling long-lived memory T cells (66).

In a recent genome-wide CRISPR KO screen of CB-NK cells, MED12, ARIH2 and CCNC were identified as regulators of CAR-NK (101). CCNC alone or the CCNC/ARIH2 double KO in CD27CAR/IL-15-NK cells showed improved expansion and persistence. The double KO reprogrammed cells toward mTORC1 activation, cell cycle progression, interleukin responsiveness and upregulated DNA repair pathways, explaining the improvement in NK expansion. This was validated in pancreatic cancer mouse models.

Inhibitory receptors from the TME, like NKG2A, drive NK cell exhaustion (10, 102, 103). NKG2A KO can reverse NK cell dysfunction (102, 103). NK cells in relapsed AML have an NKG2A exhausted phenotype, reduced cytokine production and PI3K-AKT signaling (104). NKG2A inhibition or KO reduced LAG3, TIGIT and PD-1 expression and activated AKT signaling, reversing NK cell exhaustion (104). This suggests that NKG2A KO or inhibition could improve CAR-NK persistence. Importantly, while disruption of inhibitory checkpoints can improve CAR-NK activation and effector function, it can also lead to poor expansion and persistence due to increased apoptosis (105). NKG2A disruption can enhance CAR-NK effector function but at the expense of persistence and thus, achieving an appropriate balance between activation and survival pathways, such as through the disruption of FAS, is critical (105). This co-disruption of NKG2A along with FAS helps improve the effector function with improved persistence highlighting the importance of balance of CAR-NK cell activation and survival.

A common emerging theme with deleting CISH, CREM, CCNC, ARIH2 is that the effective persistence improving strategy reprograms the fundamental cellular state of NK cells and not just the cytotoxicity. Increasing IL-15 responsiveness, metabolic fitness, mitochondrial function or memory-like transcriptional programs produce more long-lasting benefits than just a boost in effector functions. It is worth noting that several successful intracellular checkpoint disruptions converge on common pathways like JAK/STAT, mTOR and MYC, hinting the importance of metabolically rewiring CAR-NK cells might be the way to enhance persistence.

#### 3.3.2 Disruption of extrinsic checkpoints and suppressive cytokines

TGF-β is overexpressed by cancer cells and immunosuppressive cells like Tregs and MDSCs. NK cell function is impaired in HCC, a cancer with high TGF-β. Knocking out TGF-β receptor 2 (TGFBR2-KO) or expression of a dominant negative (DN) form of the TGF-β receptor 2 (TGFBR2-DN) in HCC-specific CAR iPSC-NK can improve antitumor activity and CAR-NK persistence (106). TGF-β inhibition was more important than HCC specificity in CAR-NK cells for antitumor function and persistence. TGFBR2-KO iPSC-NK cells had higher expression of activating receptors (NKG2D, DNAM-1) with higher transcription of activation genes, anti-tumor activity genes, cell proliferation genes, cytokine signaling pathways, and chemokine activation. This transcriptomic reprogramming explains improved persistence of TGFBR2-KO/DN iPSC-NK (**Figure 3B-1**). In AML patients, NK cells exhibit global dysfunction through the αvβ integrin/TGF-β/SMAD pathway, inducing TF BATF (107). BATF upregulates NK exhaustion genes like LAG3, CTLA4, TIGIT and HAVCR2. Deleting BATF improves NK cell function, and as TGF-β is upstream of BATF, CB-derived CAR.70/IL-15 transduced/TGFBR2KO NK cells are now being tested in a clinical trial against relapsed/refractory myeloid malignancies (NCT06930651).

During persistent activation, NK cells can upregulate PD-1 and mediate functional exhaustion (108). Inhibiting or disrupting the inhibitory immune checkpoint PD-1is currently under investigation (**Figure 3B-2**). Approaches for knocking out inhibitory signaling molecules using CRISPR/Cas9 in primary NK cells are well established and validated by knocking out ADAM17 and PCD1 genes (109). Data shows that increased PD-1-KO-PB-NK cells were present at endpoint in the ascites fluid in a xenograft ovarian cancer model. These KOs had superior effector function, but improvements in survival were modest. Although resting NK cells have low PD-1 expression immunosuppressive TME can upregulate PD-1 levels. A multi-arm phase I trial using 3 patients per group found that PD-1 blockade with mbIL-15-NKG2D-CAR-NK for refractory metastatic colorectal cancer increased CAR-NK cells detected at endpoint (NCT05213195) (110). Further assessments of larger cohorts are needed.

In another study, TGF-β receptor II (TRII) extracellular domain was fused to IL-21 receptor TMD (21R) (**Figure 3C**) (111). This chimeric receptor converted inhibitory TGF-β signaling into IL-21R-STAT3 activation, resulting in Bcl-2 upregulation, reduced Annexin V^+^ and cleaved caspase-3, downregulation of exhaustion markers PD-1, TIM-3, and TIGIT, and maintaining granzyme B and IFN-γ expression. TRII/21R-CAR-NK-92 cells displayed enhanced proliferation, reduced apoptosis and exhaustion within the TME, suggesting a potential survival advantage. These results were generated using non-irradiated NK-92 cells in gastric cancer models, whereas clinical translation requires irradiation to prevent malignant engraftment, impairing proliferation and limiting *in vivo* persistence.

Targeting extrinsic checkpoints protects CAR-NK cells from hostile TME. Disruption of TGF-β signaling shows great promise as it can reduce multiple downstream exhaustion programs and improve proliferation at the same time. However, benefits from disrupting PD-1 can be variable due to low baseline expression of PD-1 on resting NK cells. Depending on the disease context, the immunosuppressive TME can increase the PD-1 levels and drive poor CAR-NK persistence. Therefore, the degree to which each extrinsic checkpoint drives poor NK cell persistence may be dependent on disease context and the TME, highlighting the importance of disease specific engineering strategies to improve persistence.

#### 3.3.3 Gain-of-function engineering for signaling modulation based on genomic screening

Genome-wide loss-of-function or gain-of-function CRISPR screens have been applied to specific NK/CAR-NK cell model systems to identify novel factors that regulate/augment NK-cell functions in immunotherapy. As we described above, Biederstadt et al., has reported MED12, ARIH2 and CCNC as regulators of CAR-NK (101). Nikolic et al., identified UBE2F and ARIH2 enzymes regulate IL-15R degradation; ablation of these genes enhanced IL-15 signaling and anti-tumor immunity *in vivo* (112). More recently, Peng et al., analyzed tumor-infiltrating NK cells in an *in vivo* AAV-SB-CRISPR screen to identify *CALHM2* as a negative regulator of NK cell-cytotoxicity, degranulation and cytokine production (113). Kim et al, identified metabolite-sensing *GPR183*, *GPR84*, *GPR34* and *GPR18* as top enhancers of NK infiltration and chemotaxis to breast and ovarian cancers (114). Nguyen et al., uncovered identify regulators of cell proliferation, degranulation, and resilience to prostaglandin E2 (PGE2)-mediated immunosuppression (115).

OR7A10 a G protein-coupled receptor (GPCR) was identified as a primary human CAR-NK cell hyperbooster during an unbiased *in vivo* CRISPR activation screen (116). OR7A10 gain-of-function (GOF) CAR-NK cells had superior effector function, activation, metabolic fitness, proliferation and persistence. CB-OR7A10-CAR-NK with co-cistronic IL-15 expression had improved cytotoxicity and enhanced functional persistence in *in vitro* repeated colon tumor challenge assays when compared to the control. Although PB-OR7A10-CAR-NK cells showed superior antitumor effects compared to the control in *in vivo* ovarian cancer models, only a few NK cells were detected on day 14 across tissues. This observed CAR-NK persistence improvements might be model specific. This study is a great example of how GOF engineering combined with disruption of inhibitory mechanisms can help hyperboost NK cells with improved persistence (116) (**Figure 3D**). Although initial data suggest OR7A10 hyperboosts CAR-NK cells and improves persistence, further preclinical work is required to define the role of OR7A10 in persistence. Additionally, further long-term studies need to be done to check if this hyperboosted CAR-NK cell can be safely used in the clinic without any toxicities and accelerated differentiation.

#### 3.3.4 Multiplex checkpoint editing to disrupt intrinsic and extrinsic checkpoints

Methods for knocking out inhibitory signaling molecules using CRISPR/Cas9 in primary NK cells are well established (109). However, multiple genes may need to be edited at once as tumors can use different mechanisms to escape NK cells. To improve persistence, multiplex-engineered PB-derived CD19-CAR-NK cells were engineered with CRISPR/Cas9 base editors and non-viral TcBuster transposon-based integration (117, 118). These NK cells have a CD19-CAR with soluble IL-15, TIGIT, PDCD1 and CISH genes KO (TPCko) (**Figure 3E**) (118). CISH-KO amplifies IL-15-STAT5 signaling and metabolic fitness, while PD-1 and TIGIT-KO prevent exhaustion. IL-15-TPCko-CAR-NK had superior expansion and persistence in lymphoma xenograft models unlike IL-15 alone or TPCko alone. IL-15 was the dominant driver of persistence, with TPCko synergistically improving CAR-NK persistence and tumor control. However, excessive proliferation of IL-15-TPCko-CAR-NK caused systemic toxicities in some mice and thus, further safety optimization is required.

These findings provide insight that persistence is regulated through multiple interconnected pathways rather than one pathway as we observe superior persistence with multiplex engineering. Cytokine armoring, metabolic reprograming and checkpoint disruption have proven to be more effective than targeting an individual mechanism, however this comes with the cost of safety as multiple systemic toxicities were observed. This could be due to amplification of multiple pro survival pathways. Safety needs to be optimized in these multiplex products, incorporation of a safety switch or utilizing mbIL-15 over secreted IL-15 might lead to a more favorable safety profile.

### 3.4 Alloevasion, fratricide and Fas-FasL evasion strategies

**Figure 4:**
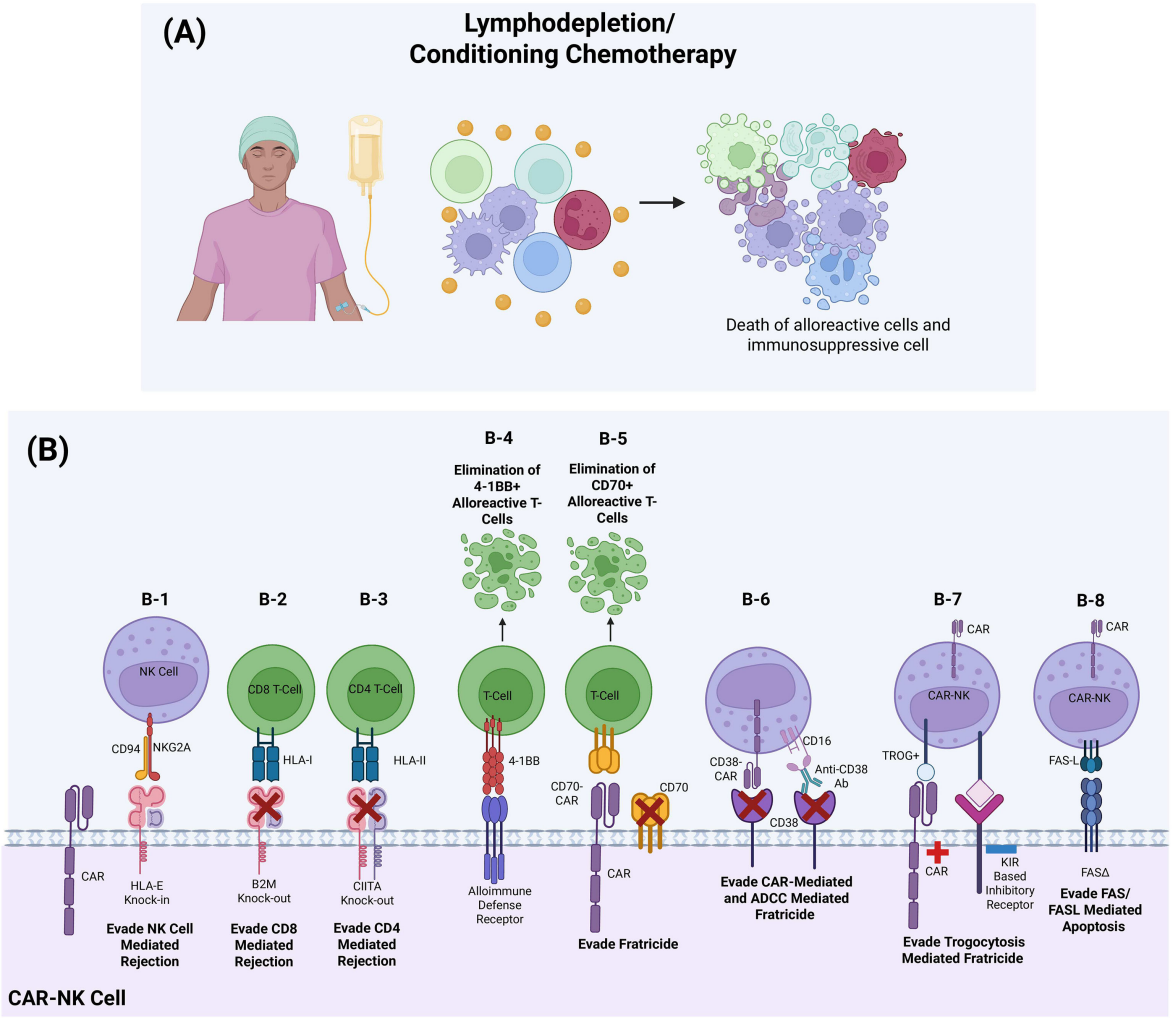
Lymphodepletion, Alloevasion, Fratricide Evasion, FAS/FASL Mediated Apoptosis Evasion Strategies Used to Improve CAR-NK Persistence. **(A)** Lymphodepletion or conditioning chemotherapy can eliminate alloreactive immune cells and immunosupressive immune cells from the body, therefore allowing adoptively transferred CAR-NK cells to persist longer. **(B)** Strategies used to evade alloreactive lymphocytes include: **(B-1)** HLA-E knockin in CAR-NK cells to evade host NK cell mediated allorejection; **(B-2)** B2M knock-out in CAR-NK cells to evade CD8 T-cell mediated allorejection; **(B-3)** Class II major histocompatibility complex transactivator, CIITA knock-out in CAR-NK cells to evade CD4 T-cell mediated allorejection; (B-4) Alloimune Defense Receptor (ADR) knock-in to selectively eliminate 4-1BB+ alloreactive T-cells; and **(B-5)** CD70-specific CAR to eliminate CD70+ alloreactive T-cells and knocking out CD70 at the same time in CAR-NK to prevent CD70 mediated fratricide. Fratricide evasion strategies utilized involve: **(B-6)** CD38 knocked out in CAR-NK cells targeting CD38 to prevent fratricide or when CD38 is being targeted through anti-CD38 antibody (Ab). **(B-7)** Trogocytosis mediated fratricide evasion strategies utilize an activating CAR-receptor specific to the tumor antigen or the trogocytosed tumor antigen (TROG+) and an inhibitory Killer cell Immunoglobulin-like Receptors (KIR) delivering a ‘do not kill me’ sign to prevent fratricide. **(B-8)** FAS/FASL mediated apoptosis/fratricide evasion stratgies utilize a dominat negative ΔFAS receptor that cannot activate the death signal. Created with BioRender.com.

Another cause for poor allogenic CAR-NK persistence is rejection by the host immune system. Lymphodepleting (conditioning) chemotherapy fludarabine and cyclophosphamide (Flu/Cy) delays rejection by reducing recipient lymphocytes and increasing serum IL-15 (119). With Flu/Cy, donor NK cells were detectable up to 14 days after transfer, showing better immunosuppression and engraftment in AML patients (119, 120). The intensity of lymphodepletion was proportional to NK cell homing to bone marrow in AML patients (120, 121). NK cell expansion was inversely proportional to pre-conditioning CD3+ T cell numbers, and adoptively transferred NK cell persistence correlated with T cell exhaustion markers (120, 122). Even autologous NK cells may benefit from lymphodepletion by depleting immunosuppressive cells and cytokine competitors, creating an environment that supports NK cell expansion and persistence (120, 123). Lymphodepletion eliminates immunosuppressive lymphocytes and improves CAR-NK persistence (**Figure 4A**) (6).

These studies highlight that in addition to engineering strategies, the effects of the host immune environment on the persistence of CAR-NK are important. A favorable environment for CAR-NK persistence is created during lymphodepletion by reducing immune rejection and increasing cytokine availability. However long-term persistence may require a combination of so engineering strategies as well as lymphoid depletion.

#### 3.4.1 Alloevasion and fratricide evasion strategies

Most current CAR-NK trials use off-the-shelf allogeneic cells but host immune recognition via HLA-I/II limits persistence and therapeutic efficacy (6). Different alloevation strategies are being explored to improve CAR-NK persistence. One approach is HLA-I and HLA-II deletion in CAR-NK cells to evade detection by CD8+ and CD4+ T cells (6). To prevent NK cell-mediated rejection, HLA-E knock-in suppresses the ‘missing-self’ response triggered by absent HLA class I (6, 124). HLA-I KO targeting Beta-2-microglobulin (B2M) and HLA-E knock-in to prevent NK cell-mediated rejection is being explored in immunotherapy (**Figure 4B-1/2**) (125). CNTY-101 is a IL-15-armored iPSC-CAR-NK with B2M and class II major histocompatibility complex transactivator, CIITA deletion (**Figure 4B-3**) to KO both HLA-I and HLA-II (126). CNTY-101 has HLA-E knock-in to prevent NK cell-mediated rejection and an EGFR knock-in for cetuximab-responsive safety switch (126). CNTY-101 showed antitumor activity and persisted up to 28 days without lymphodepletion in Phase I ELiPSE-1 trial for B-cell malignancies (NCT05336409) (127). CNTY-101 is also being tested for refractory B-cell-mediated autoimmune diseases in the CALiPSO-1 phase 1 trial (NCT06255028).

Alloimmune Defense Receptor (ADR) enables CAR-NK to eliminate 4-1BB+ alloreactive T cells (**Figure 4B-4**) (128). 4-1BB engagement eliminates alloreactive T cells while simultaneous CD3ζ signaling boosts CAR-NK activation and survival (128). In preclinical studies, ADR^+^ iPSC-derived CD19-CAR-NK cells expanded and eliminated allogenic primed T cells and tumor cells. Whereas ADR^-^ CAR-NK cells were rejected and lost tumor control. This strategy was clinically translated into iPSC-derived CD19-CAR-NK product, FT522 in BCL (NCT05950334) (129). FT522 has an ADR, IL-15/IL-15Rα, high affinity CD16, and CD38 KO. Early Phase I data show FT522 is safe, induces complete responses with delayed 4-1BB^+^ T cell recovery, and is being tested with and without lymphodepleting chemotherapy.

**Figure 5:**
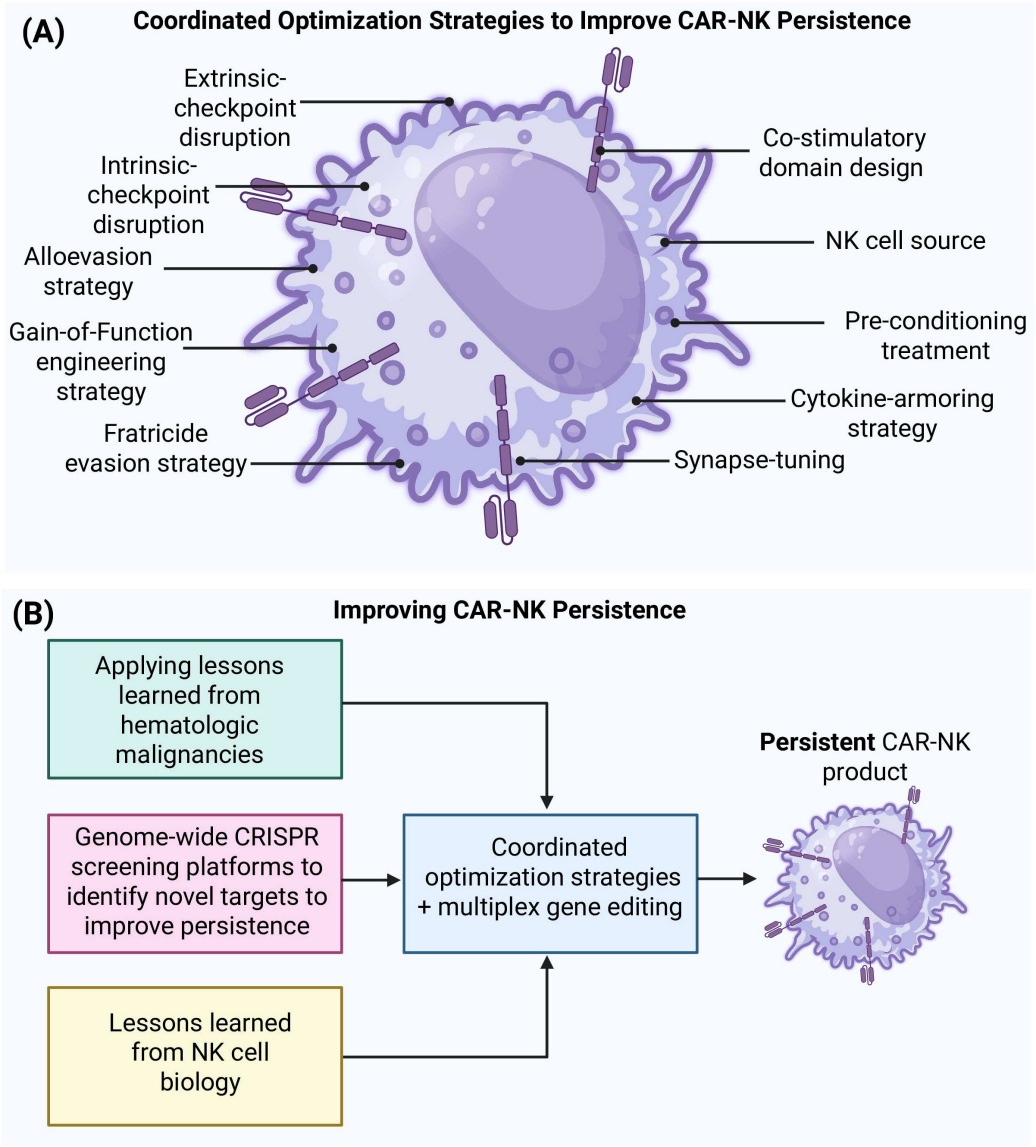
Holistic approach to improve CAR-NK persistence. **(A)** Coordinated optimization of several CAR-NK design strategies are needed to overcome the poor persistence hurdle. Next, utilizing these novel targets, and already established targets throguh multiplex gene editing of CAR-NK allows for an optimal persistent CAR-NK product. **(B)** Improving CAR-NK persistence involves, applying lessons learned from hematologic malignancies, applying lessons learned from NK cell biology and utilizing unbiased genome-wide CRISPR screens to discover novel targets to improve persistence. Created with BioRender.com.

CD8+ T cells are identified as the dominant driver of allorejection (130). To overcome CD8-mediated rejection, PB-CAR-NK cells were engineered with HLA-ABC knockdown plus PD-L1 or HLA-E overexpression. These CAR-NK cells had reduced exhaustion, evaded allorejection, and safely killed tumor cells in ovarian cancer and BCL models (130). CD70 KO iPSC-CAR-NK cells have been developed with a CD70-targeting CAR (70CAR-iNK), high-affinity non-cleavable CD16, and an IL-15/IL-15Rα fusion (131). CD70 KO prevents fratricide and CD70-CAR is utilized to eliminate CD70+ alloreactive T cells (**Figure 4B-5**) (131). 70CAR-iNK cells show robust cytotoxicity, improved survival, and *in vivo* persistence in lymphoma and renal cancer models (131).

CD38 is commonly expressed in MM. Daratumumab (DARA), an antibody targeting CD38, used to treat MM can also deplete CD38+ NK cells through ADCC leading to poor NK cell persistence. Adoptive transfer of CD38-KO PB-NK cells with DARA showed superior persistence compared to PB-NK cells with DARA (132). CD38-KO-NK cells had metabolic reprogramming with increased mitochondrial respiratory capacity. Translating this to PB-CAR-NK cells, CD38-KO cells resist fratricide and effectively target AML, and pretreating AML cells with all-trans retinoic acid increased CD38 expression and sensitized them to CAR-NK killing (133). This modification mitigates fratricide when combined with CD38-targeted monoclonal antibodies and enhances NK-cell resilience under oxidative stress in the TME (**Figure 4B-6**) (6, 134). Building on this CD38-KO iPSC-CAR-NK cells FT522 and FT576 are now being evaluated for BCL and MM in a clinical trial (NCT05950334 and NCT05182073) (6, 75, 135).

Trogocytosis also limits CAR-NK persistence. When CAR-NK cells attack tumor cells, they can absorb small pieces of tumor membrane containing the target antigen and display it on their own surface, making them appear like tumor cells to other CAR-NK cells. This triggers self-recognition and fratricide, leading to loss NK cells and reduced persistence (136). To overcome this, a dual-CAR system was designed in CB-NK cells combining an activating CAR targeting the tumor antigen with an inhibitory CAR that recognizes an NK self-antigen (**Figure 4B-7**) (136). The inhibitory CAR delivers a “don’t kill me” signal when NK cells interact with each other, preventing self-destruction. This dual-CAR approach effectively prevented trogocytosis-induced fratricide and improved CAR-NK persistence and antitumor activity in HM and ovarian cancer tumor models.

The results show that in addition to intrinsic survival limitations, fratricide and alloevation can cause premature CAR-NK cell death leading to poor persistence. The strategies discussed in 4.1-4.2 help improve persistence by enhancing CAR-NK cell fitness. On the other hand, fratricide evasion and alloevasion strategies help improve CAR-NK persistence by preventing host immune system mediated rejection and fratricide. This shows the importance of how CAR-NK products would benefit from both cellular fitness enhancement as well as protecting from immune mediated elimination and fratricide evasion to sustain long term persistence.

#### 3.4.2 FasL-FAS mediated death of CAR-NK

CAR-NK cell persistence is limited by an activation-induced FasL-FAS autoregulatory circuit (39). During repeated antigen stimulation, CAR-NK cells upregulate FAS-L, which engages FAS on neighboring CAR-NKs and triggers apoptosis, limiting CAR-NK persistence. This can be disrupted by introducing a dominant negative FAS receptor (ΔFAS). In competitive *in vivo* models, CB-derived ΔFAS CAR-NK cells outcompeted conventional CAR-NK cells, showing superior persistence without impairing cytotoxicity in a lymphoma model. The FasL-FAS pathway limits CAR-NK persistence but not the effector function and can be used to improve CAR-NK persistence (39) (**Figure 4B-8**). This is a great example where repeated antigen stimulation can drive activation induced apoptosis in CAR-NK cells, limiting its persistence. Some persistence enhancing strategies might not improve effector function but preserve the pool of functional CAR-NK infused leading to improved persistence.

## 4 Conclusion and future perspectives

Limited persistence of unmodified CAR-NK remains a major hurdle to unlocking its full therapeutic potential (9–12). Unlike CAR-T cell therapy, which often requires a single dose, repeated higher doses of CAR-NK are necessary to combat poor CAR-NK persistence (6, 137). Strategies explored to improve CAR-NK persistence include cytokine-mediated strategies, optimal NK cell sourcing, CSD tuning, checkpoint disruption, lymphodepletion, alloevasion and fratricide evasion, and disruption of Fas/FasL-mediated apoptosis. Not all persistence types are equal and CAR-NK products need to show peripheral, functional, tumor localized and safe controllable persistence in preclinical studies before it can be translated into the clinic.

### 4.1 Translating persistence strategies from hematologic malignancies to solid tumors

Cytokine-engineering strategies explored and validated in HM have now been implemented in several solid tumor clinical trials (6). CIML CAR-NK cells have been examined in both solid and hematological malignancies (50–53, 138). A key novel finding is that not all CIML NK cells have the same fate. CIML differentiation improves NK-cell persistence through epigenetic and metabolic reprogramming (139). IL-12/15/18 pre-activation of PB-NK cells with gives rise to two fates: enriched memory-like (eML) NK cells or effector conventional NK (effcNK) cells (139). eML-1 cells displayed distinct chromatin and transcriptional profiles, maintained IFN-γ production, and persisted up to 60 days post-infusion in AML patients without cytokine support, whereas effcNK cells declined rapidly. Specifically, the eML-1 subset drives long-term persistence in AML (139). Therefore, future CAR-NK strategies in HM and solid tumors should enrich this eML-1 population before CAR engineering to improve persistence. Hematopoietic growth factor signaling such as Thrombopoietin (TPO/THPO) and its receptor c-MPL, enhances CAR-NK-92 survival through JAK-STAT and PI3K-AKT pathways by upregulating pro-survival proteins (Bcl-2 and Bcl-XL) in HM (140). This preclinical work could be explored in solid tumors as a cytokine-independent strategy to improve persistence. Additionally, intrinsic checkpoint disruption (CISH-KO and CREM-KO) improves metabolic fitness and persistence of CAR-NK cells in HM. This is another promising approach to be explored in solid tumors to improve persistence.

### 4.2 Leveraging NK cell biology to improve CAR-NK persistence

NK cell telomere shortening limits NK cell proliferation and lifespan. Human telomerase reverse transcriptase (hTERT)-engineered-NK cells have higher proliferation, activation, and improved lifespan without immortalization (141). This could improve CAR-NK persistence pending safety evaluation. In TME, inhibitory receptors TIGIT, TIM-3, LAG3, and NKG2A drive NK cell exhaustion (10, 102, 103). TIGIT blockade using a monoclonal Ab can prevent NK cell exhaustion, resulting in improved colon tumor control (142). However, TIGIT blockade is context dependent. In AML, it improved cytotoxicity of exhausted NK-92 cells but not CIML NK cells, suggesting that TIGIT blockade is most effective when NK cells are exhausted (143). Fc-active anti-TIGIT antibodies may result in NK cell fratricide through CD16, however, TIGIT KO NK cells have enhanced cytotoxicity, improved metabolic fitness, and are resistant to fratricide (144). GD2 PB-CAR-NK cells with TIGIT KO have been explored (145). In a LA-N-1 neuroblastoma tumor immune microenvironment (TiME) xenograft model, they found more intratumoral GD2-CAR-NK TIGIT KO cells when compared to GD2 CAR-NK 4 days post NK cell infusion. Intratumoral unmodified PB-NK cell numbers were increased at the 4-day time point compared to TIGIT KO PB-NK cells or the CAR-NK cells in this particular model. This study shows increased TIGIT KO CAR-NK cells compared to WT at a short time point, although the importance of TIGIT in defining CAR-NK persistence is still unclear (145). LAG3+ NK cells are activated but hyporesponsive with high metabolic activity with low cytokine production and functional exhaustion driven by TOX in many cancer models (146). LAG3 could play a role in CAR-NK cell dysfunction limiting persistence and LAG3 KO or blockades could be explored to improve persistence (147, 148). Overexpression of TIM-3 leads to NK cell dysfunction and exhaustion with lowered cytokine receptors and key TFs like Eomes and T-bet (149, 150). TIM-3 blockade reverses exhaustion, suggesting TIM-3 disruption or TIM-3 KO may improve CAR-NK persistence (148–151). TIGIT, LAG3 and TIM-3 are NK inhibitory checkpoints that play a role in NK cell exhaustion. Disruption of these checkpoints improves NK cell function, however its role in improving CAR-NK cell persistence warrants further investigation.

Adenosine in the TME is an immunosuppressive metabolite that impairs NK cell function (152). Adenosine A2A receptor (A2AR) signaling is a key intrinsic checkpoint that negatively regulates NK cell maturation. A2AR-KO-NK cells had a terminally mature phenotype, improved proliferation and better tumor control in melanoma model and fibrosarcoma model, suggesting this could improve CAR-NK persistence (153).

NK cell activation can also be inhibited through imbalanced proteostasis which is triggered due to nutrient stress within the TME (154). Intra-tumoral NK cell persistence can be limited due to the transcriptional repression of the pro-survival unfolded protein response by FLI1 (154). FLI1 KO primary NK cells had lower protein aggregation, improved effector function and improved persistence in *in vivo* tumor models (154). This promising work suggests that FLI1 KO CAR-NK cells might have improved persistence, especially in regard to solid tumors.

Disruption of pro-apoptosis genes has also been explored as a strategy to improve NK cell persistence (155). NOXA (PMAIP1) is a BH3-only protein that neutralizes anti-apoptotic BCL-2 family proteins, disrupts mitochondrial integrity, inducing apoptosis. NOXA-KO PB-NK cells had improved proliferation, sustained cytotoxicity and enhanced serial killing through increased metabolic fitness (155). This preclinical research shows how targeting apoptotic pathways can be a promising strategy to help improve the persistence of CAR-NK cells (155).

Another approach that needs to be explored to improve CAR-NK cell survival is the overexpression of pro-survival proteins. IL-15 signaling can induce the expression BCL2, BCL-XL and MCL1 (156). MCL1 is essential for NK cell survival, followed by BCL2 and BCL-XL being redundant (156). However, whether anti-apoptotic protein overexpression in CAR-NK could promote the lifespan, is yet to be explored. BCL-XL overexpressing CAR-T cells have been shown to improve persistence (157).

### 4.3 Design strategies to enhance safety

Although conventional CAR-NK cell therapy is associated with relatively low levels of immune-related adverse events (38, 158, 159), safety concerns may emerge as one starts to promote persistence of CAR-NK in immunotherapy. For instances, de-regulated IL-15 expression may increase the risk of uncontrolled cellular expansion, chronic immune activation, cytokine-associated toxicity, and off-tumor tissue injury (64, 69); advanced gene-editing strategies might also associate with unintended genomic alterations (114, 160, 161), which lead to genetic instability, loss-of-function events, or even oncogenic transformation (20).

Inducible safety switches represent one of the most practical strategies, allowing selective elimination or functional suppression of infused CAR-NK cells if severe or persistent toxicity occurs. For example, the most widely used switch, inducible caspase-9 (iC9) system functions by modifying the target cell to express chaperon protein such as FKB12 fused with modified caspase 9. When the CAR-NK induced toxicity occurs, a small-molecule dimerizer drug is given; it binds FKBP domains and forces iCasp9 molecules to dimerize, which activates caspase 9 and then downstream effector caspases such as caspase 3, rapidly inducing apoptosis of the uncontrolled CAR-NK cells (162). Since the dimerizer drug has little effect on cells lacking iCasp9, this method is considered highly specified against pathogenic CAR-NK cells and has been incorporated into IL-15 expression CD19 CAR-NK products, and current clinical trials suggest low incidence of cytokine induced cytotoxicity (38, 163). In addition to iC9, other inducible control systems have also been described, such as the truncated EGFR. Briefly, the CAR construct is designed so that CAR-NK cells co-express a truncated version of human EGFR, which kept the extracellular antibody binding epitope and cell surface expression, but removes the normal ligand-binding domains and intracellular kinase signaling domains, so it should be functionally inert (164). Once the CAR-NK causes toxicity, monoclonal antibody cetuximab could be given to the patient and selectively eliminate the engineered cells (164, 165). By providing an external mechanism to terminate or modulate CAR-NK activity, inducible switches may reduce the risks of uncontrolled expansion, prolonged cytokine release, and on-target/off-tumor toxicity. However, their protective effect depends on timely clinical recognition of toxicity, adequate drug delivery, and efficient elimination of all engineered cells; therefore, inducible switches should be viewed as an important but not standalone safety solution.

The logic gate system is another emerging strategy to improve therapeutic safety by making CAR-NK activation more dependent on the combinatorial antigen pattern of tumor cells rather than recognition of a single antigen alone. For instance, the “AND” gate requires two distinct antigens to be recognized simultaneously for CAR activation (166, 167). In some design, antigen recognition and signaling are split between two receptors: one receptor provides a weakened CD3ζ activation signal after binding the first antigen, while a second chimeric costimulatory receptor provides CD28 and/or 4-1BB signaling after binding the second antigen (168). Because neither signal alone is sufficient to drive full effector function, CAR activity is preferentially restored only at sites where both antigens are co-expressed, enhancing tumor selectivity and reducing on-target off-tumor toxicity.

Meanwhile, the “AND-NOT” gate adopts inhibitory CARs that recognizes self-antigen on the healthy cells to prevent damage (169, 170), mimicking the “missing self” character of the NK cells. In general, this design incorporates an activating CAR that recognizes tumor-associated antigen and triggers cytotoxic function, and a second inhibitory CAR, or iCAR, recognizes a protective antigen expressed on normal/off-target tissues. The iCAR contains inhibitory signaling domains, such as those derived from PD-1 or CTLA-4, so that when the engineered cell encounters a target expressing both the activating antigen and the inhibitory antigen; the inhibitory signal suppresses CAR-mediated cytokine release, proliferation, and cytotoxicity (170). Overall, logic gated CAR-NK engineering provides a way to preserve antitumor potency while reducing unintended killing of normal tissues, particularly in solid tumors where target antigens are often heterogeneous and incompletely tumor specific.

Beside the two methods described, more strategies to protect the patients from the CAR-NK induced toxicity have been proposed and verified, including regulating cytokine expression, adaptor-dependent CARs, and rigorous genomic safety assessment during gene editing (171, 172). Together, these control systems provide an essential safeguard for next-generation armored CAR-NK therapies, helping to balance enhanced persistence and antitumor potency with the need for rapid clinical intervention in the event of uncontrolled expansion or treatment-related toxicity.

### 4.4 Integrated design strategies for enhancing CAR-NK persistence

Improving CAR-NK persistence depends on coordinated optimization of multiple design parameters, including NK cell source, CSD, cytokine armoring, checkpoint disruption, alloevasion, and fratricide evasion strategies (**Figure 5A**). Recent advances show that no single modification is sufficient to improve persistence but comes from integrating several synergistic edits within the same product. Multiplex editing has emerged as a promising strategy that allows simultaneous engineering strategies to be incorporated to the same CAR-NK product (118). Unbiased genome-wide CRISPR screens are instrumental in identifying novel intrinsic bottlenecks that regulate NK exhaustion, metabolic fitness, and cytokine responsiveness (101). Advances in non-viral engineering and multiplex base editing allow simultaneous KO or gain-of-function edits without double-strand breaks, with cytokine armoring, CAR insertion, and multi-checkpoint disruption within a single manufacturing step. Together, CRISPR screen platforms and multiplex editing strategies are essential to overcome the persistence barrier (101, 118).

This review highlights the causes of poor CAR-NK persistence and different strategies explored to improve persistence. To build a persistent CAR-NK product, we need to identify persistence improving targets through unbiased CRISPR screens or through lessons learned from HM, NK cell biology and fine-tune and integrate different strategies into one CAR-NK product (**Figure 5B**). Overcoming poor CAR-NK persistence and unlocking its full therapeutic potential with minimum side effects or safety concerns would improve the treatment landscape of cancers.
